# The association between adherence to the Mediterranean diet and hepatic steatosis: cross-sectional analysis of two independent studies, the UK Fenland Study and the Swiss CoLaus Study

**DOI:** 10.1186/s12916-019-1251-7

**Published:** 2019-01-24

**Authors:** Saman Khalatbari-Soltani, Fumiaki Imamura, Soren Brage, Emanuella De Lucia Rolfe, Simon J Griffin, Nicholas J Wareham, Pedro Marques-Vidal, Nita G Forouhi

**Affiliations:** 1 Medical Research Council Epidemiology Unit, University of Cambridge School of Clinical Medicine, Cambridge, CB2 0QQ UK; 2 Department of Internal Medicine, Internal Medicine, Lausanne University Hospital (CHUV), rue du Bugnon 46, 1011 Lausanne, Switzerland

**Keywords:** Mediterranean diet, Hepatic steatosis

## Abstract

**Background and aims:**

The risk of hepatic steatosis may be reduced through changes to dietary intakes, but evidence is sparse, especially for dietary patterns including the Mediterranean diet. We investigated the association between adherence to the Mediterranean diet and prevalence of hepatic steatosis.

**Methods:**

Cross-sectional analysis of data from two population-based adult cohorts: the Fenland Study (England, *n* = 9645, 2005–2015) and CoLaus Study (Switzerland, *n* = 3957, 2009–2013). Habitual diet was assessed using cohort-specific food frequency questionnaires. Mediterranean diet scores (MDSs) were calculated in three ways based on adherence to the Mediterranean dietary pyramid, dietary cut-points derived from a published review, and cohort-specific tertiles of dietary consumption. Hepatic steatosis was assessed by abdominal ultrasound and fatty liver index (FLI) in Fenland and by FLI and non-alcoholic fatty liver disease (NAFLD) score in CoLaus. FLI includes body mass index (BMI), waist circumference, gamma-glutamyl transferase, and triglyceride; NAFLD includes diabetes, fasting insulin level, fasting aspartate-aminotransferase (AST), and AST/alanine transaminase ratio. Associations were assessed using Poisson regression.

**Results:**

In Fenland, the prevalence of hepatic steatosis was 23.9% and 27.1% based on ultrasound and FLI, respectively, and in CoLaus, 25.3% and 25.7% based on FLI and NAFLD score, respectively. In Fenland, higher adherence to pyramid-based MDS was associated with lower prevalence of hepatic steatosis assessed by ultrasound (prevalence ratio (95% confidence interval), 0.86 (0.81, 0.90) per one standard deviation of MDS). This association was attenuated [0.95 (0.90, 1.00)] after adjustment for body mass index (BMI). Associations of similar magnitude were found for hepatic steatosis assessed by FLI in Fenland [0.82 (0.78, 0.86)] and in CoLaus [0.85 (0.80, 0.91)], and these were also attenuated after adjustment for BMI. Findings were similar when the other two MDS definitions were used.

**Conclusions:**

Greater adherence to the Mediterranean diet was associated with lower prevalence of hepatic steatosis, largely explained by adiposity. These findings suggest that an intervention promoting a Mediterranean diet may reduce the risk of hepatic steatosis.

**Electronic supplementary material:**

The online version of this article (10.1186/s12916-019-1251-7) contains supplementary material, which is available to authorized users.

## Introduction

Hepatic steatosis is a major cause of chronic liver disease worldwide, with prevalence estimates ranging from 25 to 45% in the general population [1]. Hepatic steatosis, usually defined as fat accumulation > 5% in hepatocytes, is the first recognisable stage for both alcoholic and non-alcoholic fatty liver disease (NAFLD). Hepatic steatosis, especially NAFLD, may lead to progressive liver fibrosis and cirrhosis and increased risk of type 2 diabetes and cardiovascular diseases [2]. NAFLD is the primary hepatic outcome of metabolic syndrome and further cardiometabolic diseases, which include insulin resistance as well as dyslipidaemia and obesity as key pathologic mechanisms according to recent scientific advances [3, 4]. Previous dietary studies reported that, compared with healthy controls, individuals with hepatic steatosis had a higher consumption of carbohydrates, saturated fat, meat, and soft drinks, along with a tendency towards a lower intake of omega-3 polyunsaturated fatty acids (PUFA) and fish [5–8]. However, there are few data concerning associations between dietary factors and overall dietary pattern with hepatic steatosis among healthy adults.

Recently, the Mediterranean diet has been recommended for the management of NAFLD by the EASL-EASD-EASO Clinical Practice Guideline [3]. Meta-analyses and systematic reviews of published evidence have supported a beneficial impact of adherence to the Mediterranean diet on overall mortality and on risks of metabolic syndrome, type 2 diabetes, and cardiovascular disease [9–11]. However, whether the Mediterranean diet is associated with risk of hepatic steatosis remains unclear. Higher adherence to the Mediterranean diet has beneficial effects on the progression of hepatic steatosis, but this evidence was derived from small-scale trials (*n* < 90 followed up for < 6 months) in patients with existing hepatic steatosis [12–17]. Among adults free from clinically manifest hepatic steatosis, evidence from three studies is available, but still inconclusive. Two cross-sectional studies reported conflicting results: one among obese Spanish adults with high cardiovascular risk (*n* = 794) reported an inverse association [18], and the other among apparently healthy Chinese adults (*n* = 332) reported a null association [19]. The Framingham Heart Study, only one longitudinal study thus far, has recently reported a significant inverse association of greater adherence to the Mediterranean diet with incident hepatic steatosis (*n* = 1521 adults over 6 years follow-up) [20].

Given the limited and inconsistent evidence, which is mainly restricted to a single study or country, we aimed to investigate the cross-sectional association of adherence to the Mediterranean diet with hepatic steatosis among middle-aged healthy adults in two independent population-based cohorts: Fenland (East England, UK) and CoLaus (Lausanne, Switzerland).

## Methods

### Study design and population

The Fenland Study is an ongoing population-based cohort of adults from general practice lists in Cambridgeshire (Cambridge, Ely, Wisbech, and surrounding villages) in the UK [21]. Overall, 12,435 adults born between 1950 and 1975 (aged 30 to 65 years at recruitment) attended baseline clinical assessments in 2005–2015. This cohort was established to investigate environmental and genetic risk factors for the development of type 2 diabetes and related metabolic disorders. Exclusion criteria were the presence of known diabetes, being pregnant, being unable to walk unaided, or having psychosis or a terminal illness. The Cambridge Local Research Ethics Committee approved the study (04/Q0108/19), and all participants provided written informed consent.

The CoLaus Study is an ongoing population-based cohort including a random sample of 6733 individuals aged 35 to 75 years in the city of Lausanne, Switzerland. Details of the study have been described previously [22]. This cohort was established in 2003 to investigate the clinical, biological, and genetic determinants of cardiovascular disease, and it included participants of European origin. For the present analysis of CoLaus, we used data from the first follow-up (study period 2009–2013; *n* = 5064) when dietary assessment was initiated. The Institutional Ethics Committee of the University of Lausanne approved the study (reference 16/03), and all participants provided written informed consent.

In this analysis of the two cohorts, we used standardised exclusion criteria to remove participants with diabetes [defined as glycated haemoglobin ≥ 48 mmol/mol, fasting plasma glucose ≥ 7.0 mmol/L, 2-h glucose ≥ 11.1 mmol/L, or use of glucose-lowering drugs], those who are pregnant, and those missing dietary data, outcome data, or key covariates. We excluded those with implausible energy intake based on sex-specific thresholds (< 500 or > 3500 kcal/day in women; and < 800 or > 4000 kcal/day in men) [23]. Participants with missing marital status data (*n* = 1506) in Fenland were retained for analyses and coded with a missing indicator variable.

### Dietary assessment

In Fenland, a self-administered, 130-item, semi-quantitative food frequency questionnaire (FFQ) was used to assess habitual dietary intake over the previous year [24]. The validity of the FFQ was previously assessed against 16-day weighed dietary record, 24-h recall, and biomarkers [25–27]. In this FFQ, the average consumption frequencies of each food item ranging from ‘never or less than once per month’ to ‘six or more times per day’ (nine categories) were provided. Total energy and macronutrient intake were estimated based on the UK food composition database [28].

In CoLaus, a self-administered, 97-item, semi-quantitative FFQ was used to assess dietary intake over the preceding 4 weeks [29], the validity of which had been assessed in nearby Canton of Geneva against 24-h recalls [30, 31]. For each item, consumption frequencies ranging from ‘less than once during the last 4 weeks’ to ‘two or more times per day’ (seven categories) were provided, in addition to the serving size (smaller, equal, or bigger) in comparison to a reference size.

### Mediterranean diet scores

We calculated three Mediterranean diet scores (MDSs). We evaluated the pyramid-based MDS (pyrMDS) [32] based on the Mediterranean dietary pyramid [33] as our primary exposure. The Mediterranean dietary pyramid was proposed by the Mediterranean Diet Foundation to be applied to both Mediterranean and non-Mediterranean countries [33]. We previously confirmed the content validity of pyrMDS in a non-Mediterranean setting, with its higher scores being associated with lower incidence of cardiovascular disease and cardiovascular and all-cause mortality in a UK population [32]. The detailed scoring method has been reported previously [32]. Briefly, a continuous score of 0 to 1 was assigned for each recommended consumption level of the 15 components of the pyramid (possible range 0–15): vegetables, legumes, and fish as healthy food groups; red meat, processed meat, potato, and sweets as unhealthy food groups; and fruits, cereals, nuts, eggs, dairy, white meat, and alcoholic beverages as items for which a moderate consumption was recommended (Additional file 1: Table S1). The second MDS was based on an algorithm proposed by Sofi and Casini [34] based on a systematic review of the published literature (literature-based MDS, LitMDS; possible range 0–18). The LitMDS accounted for 9 dietary items: vegetables, legumes, fruits and nuts, cereals, dairy, fish, meat, alcohol, and olive oil. The third MDS was based on each cohort’s sex-specific tertiles (tMDS; possible range 0–18) and accounted for the same 9 dietary items as LitMDS (Additional file 1: Table S1). We also previously tested these scores in a UK population [32]. The MDS calculation was adjusted to an energy intake of 2000 kcal/day (8.37 MJ/day) based on the residual method [23, 32].

### Ascertainment of hepatic steatosis

In Fenland, hepatic steatosis was ascertained by abdominal ultrasound, which is considered the first-line diagnostic procedure for hepatic steatosis [3]. A semi-quantitative grading system was used to define normal hepatic echotexture or mild, moderate, and severe steatosis. The images were scored retrospectively according to standardised criteria by two trained operators who were unaware of other study measures. Hepatic steatosis scoring criteria were (a) increased echotexture of the liver parenchyma (bright liver in comparison with the kidney), (b) decreased visualisation of the intra-hepatic vasculature, and (c) attenuation of the ultrasound beam. Each criterion was scored on a 4-point scale, and a cumulative liver fat score based on the sum of the scores was created (possible range 3–12) [35]. A score of ≤ 4 was classified as normal liver, 5–7 as mild steatosis, 8–10 as moderate steatosis, and ≥ 11 as severe steatosis. In our primary analysis, the mild, moderate, and severe categories were grouped together. The diagnostic accuracy of ultrasound was previously assessed against proton magnetic resonance spectroscopy, with sensitivity and specificity of 96% and 94%, respectively [36].

In both cohorts, we also evaluated fatty liver index (FLI) as an outcome, using anthropometry measures and fasting blood markers, calculated based on a logistic function including body mass index (BMI), waist circumference (WC), triglyceride (TG), and gamma-glutamyl transferase (GGT) levels as follows:

$$ \mathrm{FLI}=1/\left(1+{e}^{-\left(0.953\times \ln\ \left(\mathrm{TG}\right)+0.139\times \mathrm{BMI}+0.718\times \ln\ \left(\mathrm{GGT}\right)+0.053\times \mathrm{WC}-15.745\right)}\right) $$

FLI × 100 ranged from 0 to 100, and the presence of hepatic steatosis was defined by FLI ≥ 60, a value with sensitivity and specificity of 61% and 86%, respectively [37]. The diagnostic accuracy of FLI in comparison to ultrasonography has been reported to have an area under the receiver operating characteristic curve of 0.813 (95% CI 0.797, 0.830) [38].

In the CoLaus Study, abdominal ultrasound measurements were not available and hepatic steatosis was assessed by FLI (as above) and additionally by the ‘NAFLD liver fat score’ [39]. The NAFLD liver fat score was based on an algorithm including the presence of metabolic syndrome defined by the International Diabetes Federation criteria [40], presence of type 2 diabetes, and fasting concentrations of insulin, aspartate-aminotransferase (AST), and the AST/alanine transaminase (ALT) ratio:

$$ \mathrm{NAFLD}\ \mathrm{liver}\ \mathrm{fat}\ \mathrm{score}=-2.89+1.18\times \mathrm{metabolic}\ \mathrm{syndrome}\ \left(\mathrm{yes}/\mathrm{no}\right)+0.45\times \mathrm{type}\ 2\ \mathrm{diabetes}\ \left(\mathrm{yes}/\mathrm{no}\right)+0.15\times \mathrm{fasting}\ \mathrm{insulin}\ \left(\mathrm{mU}/\mathrm{L}\right)+0.04\times \mathrm{fasting}\ \mathrm{AST}\ \left(\mathrm{U}/\mathrm{L}\right)-0.94\times \mathrm{AST}/\mathrm{ALT} $$

Compared to proton magnetic resonance imaging, the presence of hepatic steatosis defined by a NAFLD liver fat score greater than or equal to − 0.640 had a sensitivity of 86% and a specificity of 71% [39]. In Fenland, AST levels were not available to calculate NAFLD liver fat score.

### Assessment of covariates

In both studies, socio-demographic, lifestyle, and health characteristics were collected by self-administered questionnaires. Socio-demographic data included age, sex, marital status (single, married/cohabiting, and widowed/separated/divorced), occupational social class (routine/manual and administrative/professional in Fenland; working and not working in CoLaus), and educational level (compulsory, secondary, and university). Health characteristics included the presence of metabolic syndrome and family history of diabetes. In the Fenland Study, test site (Cambridge, Ely, and Wisbech) and household income (< £20,000, £20,000–40,000, and > £40,000) were also used as covariates. Smoking status was classified as ‘never’, ‘former’, and ‘current’.

In Fenland, physical activity was assessed objectively using combined heart rate and movement sensing for over 6 days (Actiheart, CamNTech, Cambridge, UK) with individual calibration for heart rate using a treadmill test [41]. To estimate intensity time series, free-living data were pre-processed and modelled using a branched equation framework then summarised over time as daily physical activity energy expenditure (kcal/day). In CoLaus, physical activity was assessed with a validated self-administered quantitative physical activity frequency questionnaire [41, 42].

In both cohorts, body weight and height were measured with participants barefoot and in light indoor clothes; BMI was calculated as weight (kg) divided by height squared (m^2^). Waist circumference was measured by tape mid-way between the lowest rib and the iliac crest. In Fenland, body fat mass was also measured with dual-energy X-ray absorptiometry. Blood pressure was measured three times using an automated oscillometric sphygmomanometer in both cohorts, and the average of the two last measurements was used to define systolic and diastolic blood pressure.

Fasting venous blood samples were collected. In Fenland, blood samples were placed on ice, centrifuged, and stored at − 70 °C until analysis. In CoLaus, all assays were performed on the blood samples within 2 h of blood collection. In both cohorts, plasma TG, high-density lipoprotein cholesterol, and glucose were measured using standard enzymatic methods and ALT, AST (only in CoLaus), and GGT were measured using reference method as standardised by the International Federation of Clinical Chemistry.

In both cohorts, alcohol consumption was assessed by self-report number of alcoholic beverage units consumed in the preceding week, categorised as ‘abstainers’ (0 unit/week), ‘moderate’ (1–21 units/week for men, 1–14 for women), and ‘heavy’ (> 21 units/week for men, > 14 for women) drinkers. We undertook two approaches regarding alcohol consumption: one as a covariate and the second as a component of the MDS to evaluate adherence to the Mediterranean diet from which alcohol consumption was separated out as it is an established risk factor for hepatic steatosis [1].

### Statistical analysis

Statistical analyses were performed using Stata (version 14; StataCorp, College Station, TX). Descriptive statistics were presented as mean ± standard deviation (SD) for continuous variables and proportions for categorical variables. Cohen’s kappa statistics were calculated to assess the agreement between the FLI and ultrasound liver fat score in Fenland and between the FLI and NAFLD liver fat score in CoLaus.

Each of the three MDSs was modelled both categorically (quintiles) and continuously (per SD). Multivariable-adjusted Poisson regression was used to estimate the prevalence ratios (PR) and 95% confidence intervals (CI) [43] and to examine the association between hepatic steatosis (ultrasound liver fat score and FLI in Fenland; FLI and NAFLD liver fat score in CoLaus) as dependent variables and the different MDSs (pyrMDS, litMDS, and tMDS) as independent variables.

Analyses were adjusted for potential confounders including age, sex, marital status, occupational status, educational level, smoking status, energy intake, and physical activity energy expenditure (Fenland) or estimated total energy expenditure (CoLaus). Further adjustments for BMI as potential confounder were also conducted. In Fenland, the FFQ aimed at assessing habitual dietary intake across the previous year, whereas in CoLaus, it was over the past 4 weeks. Hence, to adjust for possible seasonal variation in CoLaus, the dates of dietary intake assessment were included in regression models.

A priori, we examined whether the association between adherence to the pyrMDS and hepatic steatosis varied by alcohol consumption, testing for statistical interaction by alcohol consumption and adherence to the Mediterranean diet, and we also conducted analysis stratified by alcohol consumption (abstainers, moderate, and heavy drinkers). We also assessed the influence of adjustment for both BMI and waist circumference; for body fat mass (Fenland only); for alcohol consumption (units/week); for clinical variables, including blood pressure > 130/85 mmHg (yes/no), TG level > 1.7 mmol/L (yes/no), high-density lipoprotein level < 1.29 mmol/L for men and < 1.03 mmol/L for women (yes/no), and glucose level ≥ 5.6 mmol/L (yes/no); and for family history of diabetes and metabolic syndrome (except for NAFLD liver fat score as metabolic syndrome is one of its components).

We conducted sensitivity analyses excluding the alcohol component from the pyrMDS to rule out the possible impact of alcohol on the observed association; excluding participants with BMI ≥ 30 kg/m^2^; excluding participants with excessive alcohol consumption; including participants with probable implausible energy intake; excluding participants with probable secondary causes of hepatic steatosis such as hepatitis B or C, HIV, or hepatotoxic medications; or including participants with diabetes (only for NAFLD liver fat score in CoLaus). The British National Formulary codes were used to identify hepatitis B and C or HIV in the Fenland cohort and the Anatomical Therapeutic Chemical classification of the World Health Organization to identify hepatotoxic medications in the CoLaus Study. Finally, we assessed the robustness of the results when modelling ultrasound liver fat score (1) using 7 as the cut-point to define hepatic steatosis (normal/mild vs. moderate/severe), and (2) continuously, we also examined the association of adherence to the Mediterranean diet with ALT and GGT levels as crude markers of hepatic steatosis [1, 44]. The latter were natural log transformed prior to regression analysis.

Possible interactions between the different MDSs with age, sex, and BMI in the main model were examined using the Wald test. We prespecified stratified analyses if significant interaction was identified. Statistical significance was considered for a two-sided test with a *p* value < 0.05.

## Results

### Characteristics of included participants

Of the 12,435 participants in the Fenland Study, 2790 (22.4%) were excluded, leaving 9645 participants (54.4% women; 48.9 ± 7.4 years) for analysis. Of the 5064 participants in CoLaus, 1107 (21.8%) were excluded, leaving 3957 participants (56.8% women; 57.0 ± 10.4 years) for analysis. The reasons for exclusion are summarised in Additional file 1: Figure S1, and the characteristics of included and excluded participants are shown in Additional file 1: Table S2. The mean pyrMDS score was 9.07 ± 1.43 and 8.45 ± 1.24 in Fenland and CoLaus, respectively. In both cohorts, adherence to the Mediterranean diet was higher among women compared to men, positively correlated with socio-economic status and negatively correlated with TG, liver enzyme levels, BMI, waist circumference, prevalence of current smokers, and prevalence of the metabolic syndrome (Table 1).

**Table 1:** Characteristics of participants by adherence to the Mediterranean diet, Fenland and CoLaus studies*

Characteristic	Fenland Study (*n* = 9645)					CoLaus Study (*n* = 3957)				
	Q1 (3.30–7.84)*	Q2 (7.85–8.73)	Q3 (8.74–9.46)	Q4 (9.47–10.28)	Q5 (10.29–14.03)	Q1 (1.83–7.45)	Q2 (7.46–8.18)	Q3 (8.19–8.82)	Q4 (8.83–9.47)	Q5 (9.48–12.18)
Age, years	48.8 ± 7.5	48.9 ± 7.3	48.9 ± 7.5	48.9 ± 7.6	49.1 ± 7.5	58.2 ± 10.7	58 ± 10.5	57.4 ± 10.4	56.3 ± 10.5	55.1 ± 9.4
Women, %	35.5	48.1	57.5	62.0	69.4	43.1	54.5	60.1	60.9	65.5
Marital status, %^†^										
Single	7.5	7.5	5.4	6.9	8.9	17.3	12.9	14.4	17.3	15.0
Married/cohabiting	66.5	68.7	70.9	70.2	69.1	54.3	57.1	60.1	59.8	58.2
Separated	7.3	7.7	7.9	7.9	9.5	28.4	30.0	25.5	22.9	26.8
Occupation, %										
Managerial or professional	42.5	54.6	61.3	66.5	73.8	–	–	–	–	–
Employed	–	–	–	–	–	53.4	56.9	55.7	63.1	67.9
Education, %^†^										
Compulsory	27.8	22.6	18.6	14.0	9.2	18.8	16.6	15.4	13.4	11.0
Secondary	55.1	50.7	46.3	41.1	36.5	65.0	64.6	61.2	58.8	59.8
University	17.2	26.7	35.1	44.9	54.3	16.2	18.8	23.4	27.8	29.2
Annual income, %^†^										
< £20,000	16.9	13.6	11.7	11.4	9.6	–	–	–	–	–
£20,000–40,000	41.7	35.3	34.2	30.0	27.5	–	–	–	–	–
> £40,000	38.7	48.3	52.5	56.6	61.0	–	–	–	–	–
Current smoker, %	20.0	11.4	10.4	8.8	6.2	26.4	22.8	21.0	18.1	16.4
Alcohol intake, unit/week^‡^										
Abstainers	4.1	4.3	4.5	3.9	2.6	21.1	23.3	24.0	24.5	24.1
Moderate	60.5	61.6	60.7	60.8	60.7	62.5	65.9	69.9	70.8	72.9
Heavy	22.7	25.9	27.5	27.7	29.5	16.4	10.9	6.1	4.7	2.9
Energy intake, kcal/day	2120 ± 599	1949 ± 595	1888 ± 550	1827 ± 531	1824 ± 527	1853 ± 633	1804 ± 628	1804 ± 606	1778 ± 588	1709 ± 583
Protein, % energy	17.6 ± 3.7	18.2 ± 3.5	18.3 ± 3.6	18.2 ± 3.5	18.0 ± 3.4	15.6 ± 3.6	15.8 ± 3.6	15.5 ± 3.1	15.5 ± 3.1	14.9 ± 2.8
Carbohydrate, % energy	46.6 ± 7.3	47.6 ± 6.8	48.2 ± 6.8	49.0 ± 6.9	49.3 ± 6.8	45.1 ± 9.7	44.9 ± 9.1	46.7 ± 8.4	47.0 ± 8.3	48.3 ± 8.0
Fat, % energy	35.3 ± 5.7	33.9 ± 5.5	33.1 ± 5.6	32.6 ± 5.7	32.6 ± 5.8	33.3 ± 6.7	34.7 ± 6.7	34.6 ± 6.7	34.6 ± 6.6	34.5 ± 6.8
PAEE, kcal/day	1048 ± 903	878 ± 750	777 ± 668	740 ± 582	696 ± 515					
TEE, kcal/day	–	–	–	–	–	2748 ± 672	2667 ± 642	2647 ± 621	2615 ± 581	2665 ± 615
Metabolic syndrome, %^§^	42.5	37.8	31.9	29.7	22.6	39.5	37.5	24.0	28.8	26.0
BMI, kg/m^2^	27.5 ± 4.6	27.2 ± 4.7	26.6 ± 4.5	26.1 ± 4.3	25.2 ± 4.2	26.4 ± 4.4	26.0 ± 4.4	25.6 ± 4.1	25.4 ± 4.2	25.0 ± 4.1
Waist circumference, cm	94.5 ± 13	92.0 ± 12.9	89.4 ± 12.5	87.9 ± 12.1	85 ± 11.9	93.4 ± 12.7	91.4 ± 12.2	89.7 ± 11.7	89.1 ± 12.0	87.8 ± 11.8
Triglycerides, mmol/L	1.3 ± 0.9	1.2 ± 0.8	1.1 ± 0.7	1.1 ± 0.6	1.0 ± 0.6	1.4 ± 0.9	1.4 ± 0.8	1.2 ± 0.7	1.2 ± 0.9	1.2 ± 0.7
GGT, U/L	39.6 ± 40.8	36.2 ± 34.4	32.5 ± 25	31.9 ± 25.2	28.5 ± 22.6	45.3 ± 72.1	35.3 ± 33.2	30.8 ± 28	30.9 ± 34.7	28.6 ± 31.1
ALT, U/L	30.8 ± 16.7	29.1 ± 15.7	27.7 ± 14.6	27.2 ± 15.6	26.1 ± 16.3	28.9 ± 22.3	26.9 ± 15.4	26.1 ± 13.8	24.8 ± 12.8	25.3 ± 14.8
AST, U/L	–	–	–	–	–	30.3 ± 14.8	28.7 ± 12.3	28.0 ± 8.7	27.5 ± 9.3	27.2 ± 7.6

*PAEE* physical activity energy expenditure, *TEE* total energy expenditure, *BMI* body mass index, *iqr* interquartile range, *GGT* gamma-glutamyl transferase, *ALT* alanine aminotransferase, *AST* aspartate aminotransferase

*In each cohort, participants were categorised into five groups by quintiles (Q1 to Q5) of the Mediterranean diet score representing the levels of dietary adherence to the Mediterranean diet pattern (defined by the Mediterranean diet pyramid) (possible range 0 to 15). A range of the points in each category is presented. Data are mean ± SD for continuous variables or percent for categorical variables, unless otherwise stated

^†^Due to some missing data, numbers do not always add to 100%. ‘Separated’ included divorced or widowed adults

^‡^Alcohol consumption categorised as ‘abstainers’ (0 unit/week), ‘moderate’ (1–21 units/week for men, 1–14 for women), and ‘heavy drinkers’ (> 21 units/week for men, > 14 for women)

^§^Metabolic syndrome defined according to the International Diabetic Federation

### Adherence to the Mediterranean diet and prevalence of hepatic steatosis

In Fenland, the prevalence of hepatic steatosis was 23.9% and 27.1% based on ultrasound and FLI, respectively. Diagnoses using FLI and ultrasound liver fat score were concordant (kappa = 0.79). In CoLaus, the prevalence of hepatic steatosis was 25.3% and 25.7% based on FLI and NAFLD score, respectively, with high concordance (kappa = 0.83) between the measures. Hepatic steatosis was more prevalent among men; those with higher BMI, waist circumference, and liver enzymes; and those with metabolic syndrome (Additional file 1: Table S2).

In Fenland, the multivariable-adjusted analysis showed an inverse association between pyrMDS quintiles and hepatic steatosis based on ultrasound (*p*_trend_ < 0.001) (Table 2), but the association was attenuated after further adjustment for BMI (*p*_trend_ < 0.043). An inverse association was also seen per one SD difference in pyrMDS [PR of 0.86 (95% CI 0.81–0.90)] but was attenuated after adjustment for BMI [0.95 (0.90, 1.00)].

**Table 2:** Association between adherence to the Mediterranean diet and prevalence of hepatic steatosis defined by abdominal ultrasound, Fenland Study

	Prevalence ratio (95% CI) across quintiles of pyramid-based Mediterranean diet score^*^					*p*_trend_	Prevalence ratio (95% CI) per SD difference^*^
	Q1	Q2	Q3	Q4	Q5		
Range of scores	3.30–7.84	7.85–8.73	8.74–9.46	9.47–10.28	10.29–14.03		
*N* total	1929	1929	1929	1929	1929		
*N* cases (score ≥ 5 of 3 to 12)	602	547	450	391	313		
Multivariable + SES + dietary factor^†^	1.00 (ref.)	0.99 (0.86, 1.12)	0.82 (0.71, 0.95)	0.76 (0.65, 0.88)	0.67 (0.56, 0.78)	< 0.001	0.86 (0.81, 0.90)
Multivariable + BMI^‡^	1.00 (ref.)	1.02 (0.89, 1.16)	0.94 (0.81, 1.08)	0.90 (0.77, 1.04)	0.88 (0.75, 1.04)	0.043	0.95 (0.90, 1.00)

*SES* socio-economic status, *BMI* body mass index

^*^In categorical analysis, the population was divided into five groups by quintiles (Q1–Q5) of the Mediterranean diet score, standard deviation is 1.43 for the Mediterranean diet score

^†^Adjusted for age (years), test sites (Cambridge, Ely, and Wisbech), sex, marital status (single, married, and divorced/widowed), occupational status (routine and professional jobs), education level (compulsory, secondary, and university), household income (< £20,000, £20,000–40,000, and > £40,000), smoking status (never, former, and current), energy intake (kcal/day), and physical activity energy expenditure (kcal/day)

^‡^Further adjusted for BMI. Results of further adjustment for waist circumference were broadly in line with of the further adjustment for BMI (data not shown)

In both cohorts, when FLI was the outcome, there was an inverse association between pyrMDS quintiles and hepatic steatosis (*p*_trend_ < 0.001) (Table 3), but the association attenuated after adjustment for BMI (*p*_trend_ = 0.001 and 0.009 for Fenland and CoLaus, respectively). Significant inverse association was seen per one SD difference in the pyrMDS in both cohorts; the observed association was attenuated but remained significant in Fenland after adjustment for BMI (Table 3).

**Table 3:** Association between adherence to the Mediterranean diet and prevalence of hepatic steatosis, Fenland and CoLaus studies*

	Prevalence ratio (95% CI) across quintiles of pyramid-based Mediterranean diet score^†^					*p*_trend_	Prevalence ratio (95% CI) per SD difference^†^
	Q1	Q2	Q3	Q4	Q5		
Fenland Study							
Range of scores	3.30–7.84	7.85–8.73	8.74–9.46	9.47–10.28	10.29–14.03		
*N* total	1929	1929	1929	1929	1929		
FLI, median (iqr)^‡^	47.6 (21.1, 76.4)	39.6 (16.1, 69.7)	30.6 (11.9, 61.3)	25.3 (10.3, 55.4)	16.9 (7.5, 42.2)		
*N* cases (score ≥ 60)	773	632	496	424	285		
Multivariable + SES + dietary factor^§^	1.00 (ref.)	0.94 (0.84, 1.06)	0.81 (0.71, 0.92)	0.75 (0.65, 0.86)	0.52 (0.44, 0.62)	< 0.001	0.82 (0.78, 0.86)
Multivariable + BMI^‖^	1.00 (ref.)	1.00 (0.88, 1.12)	0.98 (0.86, 1.12)	0.94 (0.82, 1.08)	0.76 (0.64, 0.89)	0.001	0.94 (0.89, 0.98)
CoLaus Study							
Range of scores	1.83–7.45	7.46–8.18	8.19–8.82	8.83–9.47	9.48–12.18		
*N* total	792	791	792	791	791		
FLI, median (iqr)‡	43.0 (19.0, 72.8)	37.2 (14.9, 66.5)	30.9 (13.2, 56.5)	27.4 (11.7, 53.7)	22.5 (9.4, 49.0)		
*N* cases (score ≥ 60)	297	240	170	160	135		
Multivariable + SES + dietary factor^§^	1.00 (ref.)	0.93 (0.77, 1.12)	0.74 (0.60, 0.90)	0.73 (0.59, 0.90)	0.61 (0.49, 0.77)	< 0.001	0.85 (0.80, 0.91)
Multivariable + BMI^‖^	1.00 (ref.)	1.06 (0.87, 1.28)	0.90 (0.74, 1.11)	0.85 (0.69, 1.05)	0.79 (0.63, 1.00)	0.009	0.94 (0.88, 1.006)
NAFLD liver fat score, median (iqr)^**^	− 1.5 (− 2.3, − 0.2)	− 1.6 (− 2.3, − 0.4)	− 1.7 (− 2.4, − 0.7)	− 1.8 (− 2.4, − 0.8)	− 2.0 (− 2.5, − 0.8)		
*N* cases (score ≥ − 0.640)	257	240	181	168	172		
Multivariable + SES + dietary factor^§^	1.00 (ref.)	1.03 (0.86, 1.25)	0.84 (0.69, 1.03)	0.82 (0.67, 1.01)	0.85 (0.69, 1.05)	0.022	0.93 (0.87, 0.99)
Multivariable + BMI^‖^	1.00 (ref.)	1.08 (0.89, 1.30)	0.92 (0.75, 1.12)	0.88 (0.72, 1.09)	1.02 (0.82, 1.26)	0.50	0.98 (0.91, 1.04)

*FLI* fatty liver index, *iqr* interquartile range, *SES* socio-economic status, *BMI* body mass index, *NAFLD* non-alcoholic fatty liver disease

*For this analysis, the outcome was assessed by fatty liver index and NAFLD liver fat score

**Calculated based on an algorithm including presence of the metabolic syndrome and type 2 diabetes and concentrations of fasting serum insulin, fasting serum aspartate-aminotransferase (AST), and AST/alanine-aminotransferase ratio

^†^In categorical analysis, the population was divided into five groups by quintiles (Q1-Q5) of the Mediterranean diet score, standard deviation is 1.43 and 1.24 for pyramid-based Mediterranean diet score in the Fenland and CoLaus studies, respectively

^‡^Calculated based on an algorithm including body mass index, waist circumference, triglycerides, and gamma-glutamyl transferase

^§^Adjusted for age (years), sex, marital status (single, married/cohabiting, and divorced/widowed), occupational status (routine and professional jobs in the Fenland Study and working and not working in the CoLaus Study), education level (compulsory, secondary, and university), smoking status (never, former, and current), energy intake (kcal/day), physical activity energy expenditure (kcal/day, in the Fenland Study), total energy expenditure (kcal/day—in the CoLaus Study), and date of dietary assessment (in the CoLaus Study)

^‖^Further adjusted for BMI. Results of further adjustment for waist circumference were broadly in line with of the further adjustment for BMI (data not shown)

In CoLaus, when NAFLD liver fat score was the outcome, there was a non-significant inverse association between pyrMDS quintiles and hepatic steatosis (*p*_trend_ = 0.022). Using pyrMDS as a continuous variable, there was an inverse association per one SD difference [0.93 (0.87–0.99)], which attenuated after adjustment for BMI (Table 3).

Associations using LitMDS or tMDS as exposure variables were largely consistent with those of pyrMDS (Additional file 1: Table S3).

### Other analyses

In both cohorts, analyses stratified by alcohol consumption showed that the inverse association between pyrMDS quintiles and hepatic steatosis was non-significant among ‘abstainers’ but significant among ‘moderate’ drinkers, except for NAFLD liver fat score in CoLaus. Among ‘heavy’ drinkers, the results were inconsistent across the two cohorts (Additional file 1: Table S4). Further adjustment for BMI attenuated the inverse associations between pyrMDS and hepatic steatosis within alcohol consumption strata.

Adjusting for both BMI and waist circumference attenuated the associations (*p*_trend_ > 0.05), except for FLI score in both studies (Additional file 1: Table S5). In Fenland, the inverse association between pyrMDS and hepatic steatosis as assessed by ultrasound remained significant after adjusting for body fat mass (Additional file 1: Table S5). Further adjustment for other potential confounders or mediators including alcohol consumption with and without adiposity measures, clinical variables, and presence of metabolic syndrome partly attenuated the associations (Additional file 1: Table S5). The results were not materially different when excluding the alcohol component from the pyrMDS; when excluding participants based on high BMI ≥ 30 kg/m^2^, excessive alcohol consumption, or secondary causes of hepatic steatosis; or when including participants with implausible energy intakes or with diabetes (Additional file 1: Table S5).

In Fenland, the use of a different dichotomisation of the ultrasound liver fat score weakened the association between pyrMDS quintiles and hepatic steatosis (Additional file 1: Table S6). The association per SD of pyrMDS was not altered, but CIs were widened (Additional file 1: Table S6). Associations modelling ultrasound liver fat score as a continuous outcome variable attenuated the inverse association, but it remained statistically significant (*p*_trend_ < 0.001) (Additional file 1: Table S6). Adherence to the Mediterranean diet was inversely but not significantly associated with ALT and GGT (Additional file 1: Table S7).

Finally, there were no significant interactions between adherence to the Mediterranean diet and age or sex in either cohort. A significant interaction between pyrMDS and BMI was found in both cohorts (*p*_interaction_ < 0.05), except when considering ultrasound liver fat score as an outcome in Fenland (*p*_interaction_ = 0.33); hence, due to inconsistent results, we conducted an analysis stratified by BMI categories (underweight/normal, overweight, and obese) (Additional file 1: Table S8).

In Fenland, the inverse association between pyrMDS quintiles and hepatic steatosis was non-significant (*p*_trend_ > 0.05) in the analyses stratified by BMI, except among overweight participants when considering FLI as an outcome (*p*_trend_ < 0.001). In CoLaus, considering FLI as an outcome, a non-significant inverse association between pyrMDS quintiles and hepatic steatosis was found among underweight/normal (*p*_trend_ = 0.006) and overweight participants (*p*_trend_ = 0.023). In contrast, considering NAFLD liver fat score as an outcome in CoLaus, stratified analyses showed no significant association between pyrMDS quintiles and hepatic steatosis (*p*_trend_ > 0.05).

## Discussion

In two independent population-based cohorts in the UK and Switzerland, we found an inverse association between greater adherence to the Mediterranean diet and the prevalence of hepatic steatosis. This association was consistent across three different definitions of adherence to the Mediterranean diet and with different non-invasive criteria for assessing hepatic steatosis but was attenuated when we included measures of adiposity as a covariate.

### Comparison with other studies

Our findings add to the limited number of studies that evaluated the associations between single food groups or other dietary patterns and hepatic steatosis. A cross-sectional study previously reported that an eating pattern high in alcohol and meat intakes and with a low tea consumption was associated with higher liver fat content among 354 individuals from the PopGen cohort in Germany [45]. Another cross-sectional study among Israeli adults reported a positive association between soft drink consumption and risk of NAFLD, whereas higher intake of omega-3 polyunsaturated fatty acids was negatively associated with the risk of NAFLD [5]. Additionally, one Australian prospective study among adolescents indicated that a Western dietary pattern characterised by a high consumption of takeaway foods, confectionary, red or processed meat, refined grain, chips, full-fat dairy, and soft drinks at age 14 was associated with the presence of NAFLD at age 17 years [46]. Our findings for the Mediterranean diet are consistent with a recent secondary cross-sectional analysis of the PREDIMED trial in Spain, although that study focused on overweight and obese participants at high cardiovascular risk, and assessed a dietary inflammatory index in conjunction with adherence to the Mediterranean diet [18]. Moreover, our results are in agreement with the only prospective study to date reported a significant inverse association between adherence to the Mediterranean diet and incident hepatic steatosis among healthy individuals without hepatic steatosis [20].

Nevertheless, our findings of an inverse association between Mediterranean diet and hepatic steatosis were not consistent with those from other studies. Possible explanations may include differences in population and methods. For instance, one cross-sectional study of healthy Chinese adults in Hong Kong reported no significant association between higher adherence to the Mediterranean diet and NAFLD prevalence even in the analyses without BMI adjustment [19]. This could be because the authors defined MDS based on population-specific medians of the study population, which might be too crude [19, 32]. Additionally, a small case-control study conducted in Athens compared 73 patients with NAFLD with 58 healthy controls and found no association between greater MDS and lower likelihood of having NAFLD [47].

In our study, the inverse association between adherence to the Mediterranean diet and hepatic steatosis was no longer significant after adjustment for adiposity (BMI and waist circumference) suggesting that adiposity may be a confounder or a mediator of the association. While our study was not designed to distinguish between these two phenomena, our findings were consistent with previous reports of non-significant associations between adherence to the Mediterranean diet and NAFLD after adjustment for BMI or abdominal fat [19, 47]. Our findings are also in agreement with a previous prospective study suggesting that the Western dietary pattern acts predominantly on hepatic steatosis incidence via the obesity pathway [46].

Previous studies have reported positive [48] or null [49] associations between the Mediterranean diet and liver enzymes. The null associations reported previously are in line with our results; the possible explanation could be the normal levels of liver enzymes in the majority of individuals with hepatic steatosis [50].

### Possible mechanisms and implications

The potential mechanisms explaining the inverse association of the individual components of the Mediterranean diet on hepatic steatosis have been reviewed previously [51, 52]. Adherence to the Mediterranean diet is associated with high antioxidant capacity and anti-inflammatory and anti-fibrotic effects due to high fruit and vegetable consumption [53]. The Mediterranean diet is also associated with high levels of monounsaturated fatty acids (MUFA), mostly from olive oil intake. In animal models, high MUFA from olive oil and/or PUFA improved lipid profile, decreased cytokine expression in visceral adipocytes, and decreased liver enzymes and hepatic TGs, thus attenuating hepatic steatosis [54–56]. These results have been corroborated in a meta-analysis of human studies, which confirmed that omega-3 fatty acids were negatively associated with hepatic steatosis [57].

Although epidemiological studies linking Mediterranean diet to hepatic steatosis are scarce, there is strong evidence regarding the inverse association between adherence to the Mediterranean diet and cardiovascular disease, the metabolic syndrome, and its components [9, 10, 58]. Few observational studies and randomised clinical trials have evaluated the impact of adherence to the Mediterranean diet on hepatic steatosis progression [12, 48, 49, 59]. All these studies, despite their difference in study design, sample population, outcome measure assessment methods, and various Mediterranean diet-scoring methods, reported the beneficial impact of greater adherence to the Mediterranean diet on reduced progression of hepatic steatosis. Thus, the Mediterranean diet may represent an alternative therapy for hepatic steatosis, given the well-recognised problems of achieving sustainable weight loss, which is currently the primary treatment for hepatic steatosis through lifestyle therapy involving diet and exercise. Therefore, the clinical importance of the Mediterranean diet for the prevention of hepatic steatosis deserves further discussion.

### Strengths and limitations

Our study in two population-based cohorts in two independent settings of the UK and Switzerland, together with the large sample size of nearly 14,000 participants, allowed us to conduct stratified analysis with adequate statistical power. The application of three different scoring algorithms for the Mediterranean diet enhanced the utility of representing adherence to the diet in different epidemiological settings. Finally, the consistency of the findings in two independent cohorts suggests that the results may be reasonably generalisable to other populations.

Our study also has some limitations. First, its cross-sectional design precludes any causal interpretation of the association between adherence to the Mediterranean diet and hepatic steatosis. The study design was also limited by its inability to distinguish whether adiposity mediated or confounded the observed association. Second, hepatic steatosis was based on different indicators and not on liver biopsy, which is considered to be the gold standard technique. However, liver biopsy is an invasive method, which cannot be implemented in large-scale epidemiological studies with predominantly healthy participants. In the Fenland Study, hepatic steatosis was based on ultrasound, a valid method for detecting hepatic steatosis [36], supplemented with an algorithm-based FLI, while in the CoLaus Study, two hepatic steatosis indices were used. These validated markers of hepatic steatosis are based on non-invasive and easily ascertained measurements which make them suitable for large epidemiological studies [38, 39, 60, 61], and the results were consistent irrespective of the marker used. Third, excluding participants with diabetes might have led to an underestimation of hepatic steatosis prevalence as assessed by NAFLD liver fat score and its association with the Mediterranean diet. However, findings were similar upon including participants with diabetes in the sensitivity analysis. Fourth, the methods for measuring variables (e.g. physical activity) differed between cohorts; however, the consistency of our findings suggests that the impact of the differing methods on results was minimal. Fifth, self-reported dietary intake and covariates are prone to measurement errors but the instruments used were validated against reference methods, and we found consistency of our estimates across the levels of adjustments.

## Conclusion

Greater adherence to the Mediterranean diet was associated with lower likelihood of hepatic steatosis, and this association was largely related to markers of adiposity. These cross-sectional findings were strengthened by their consistency in two independent settings in the UK and Switzerland and are hypothesis generating for the role of the Mediterranean diet in hepatic steatosis in non-Mediterranean settings. The cross-sectional findings from our population-based cohorts warrant further interventional or observational studies to test whether the improvement of adherence to the Mediterranean diet is efficacious and effective in diverse settings for the primary prevention of hepatic steatosis.

## Additional file

Additional file 1: **Figure S1.** Flow chart for the Fenland and CoLaus sample selection. **Table S1.** Mediterranean diet score specified by three definitions and their components. **Table S2.** Characteristics of participants included and excluded from the analysis, Fenland and CoLaus studies. **Table S3.** Association between adherence to the literature-based and tertile-based Mediterranean diet and prevalence of hepatic steatosis, Fenland and CoLaus studies. **Table S4.** Association between adherence to the Mediterranean diet and prevalence of hepatic steatosis within alcohol consumption strata, Fenland and CoLaus studies. **Table S5.** Sensitivity analyses for the association between adherence to the Mediterranean diet and prevalence of hepatic steatosis, Fenland and CoLaus studies. **Table S6.** Association between adherence to the Mediterranean diet and prevalence of hepatic steatosis, Fenland Study. **Table S7.** Association between adherence to the Mediterranean diet and ALT and GGT, Fenland and CoLaus studies. **Table S8.** Association between adherence to the Mediterranean diet and prevalence of hepatic steatosis within BMI strata, Fenland and CoLaus studies. (DOCX 124 kb)

## Abbreviations

ALT: Alanine transaminase
AST: Aspartate-aminotransferase
BMI: Body mass index
FFQ: Food frequency questionnaire
FLI: Fatty liver index
GGT: Gamma-glutamyl transferase
LitMDS: Literature-based Mediterranean diet scores
MDS: Mediterranean diet scores
MUFA: Monounsaturated fatty acids
NAFLD: Non-alcoholic fatty liver disease
PUFA: Polyunsaturated fatty acids
pyrMDS: Pyramid-based Mediterranean diet scores
TG: Triglyceride
tMDS: Tertile-based Mediterranean diet scores
WC: Waist circumference
