# Supplementary material for: The association between adherence to the Mediterranean diet and hepatic steatosis: cross-sectional analysis of two independent studies, the UK Fenland Study and the Swiss CoLaus Study
Source: BMC Med. 2019 Jan 24;17:19. doi: 10.1186/s12916-019-1251-7 (PMC6345041; doi:10.1186/s12916-019-1251-7)
Supplement: Supplementary file 1 — Figure S1. Flow chart for the Fenland and CoLaus sample selection. Table S1. Mediterranean diet score specified by three definitions and their components. Table S2. Characteristics of participants included and excluded from the analysis, Fenland and CoLaus studies. Table S3. Association between adherence to the literature-based and tertile-based Mediterranean diet and prevalence of hepatic steatosis, Fenland and CoLaus studies. Table S4. Association between adherence to the Mediterranean diet and prevalence of hepatic steatosis within alcohol consumption strata, Fenland and CoLaus studies. Table S5. Sensitivity analyses for the association between adherence to the Mediterranean diet and prevalence of hepatic steatosis, Fenland and CoLaus studies. Table S6. Association between adherence to the Mediterranean diet and prevalence of hepatic steatosis, Fenland Study. Table S7. Association between adherence to the Mediterranean diet and ALT and GGT, Fenland and CoLaus studies. Table S8. Association between adherence to the Mediterranean diet and prevalence of hepatic steatosis within BMI strata, Fenland and CoLaus studies. (DOCX 124 kb) [file 12916_2019_1251_MOESM1_ESM.docx]

# **Figure S1** Flow chart for the Fenland and CoLaus Studies sample selection.

Presence of diabetes (n = 322, 2.6%) * or being pregnant (n = 5, 0.04%)

Total analytic sample (n=9645, 77.56%)

Missing dietary data (n = 16, 0.13%), covariate data (n = 250, 2.0%) and outcome data (n = 2019, 16.2%)

Energy intake outliers (n = 178, 1.4%) †

Total original sample (n = 12435)

Presence of diabetes (n = 539, 10.6%) *

Energy intake outliers (n = 95, 1.9%) †

Total analytic sample (n = 3957, 78.1%)

Missing dietary data (n = 378, 7.5%), covariate data or outcome data (n = 95, 1.9%)

Total original sample (n = 5064)

**Fenland Study**

**CoLaus Study**

* Diabetes was defined as glycated haemoglobin ≥48 mmol/mol and/or plasma glucose ≥7.0 mmol/L and/or 2-hour glucose≥11.1 mmol/L with or without presence of glucose lowering drugs or insulin.

† Energy intake outliers defined as <500 and <800 kcal or >3500 or >4000 kcal in women and men, respectively.

**Table S1** Mediterranean diet score and components.

| Food groups | Mediterranean diet score (score range) | | |
| --- | --- | --- | --- |
|  | PyrMDS, based on the Mediterranean diet pyramid  (0-15) | LitMDS, based on published literature  (0-18) | tMDS, based on tertiles of dietary intakes  (0-18) |
| Vegetables | 0 to 1 | 0, 1, or 2 | 0, 1, or 2 |
| Legumes | 0 to 1 | 0, 1, or 2 | 0, 1, or 2 |
| Fruits*^*^* | 0 to 1  0 to 1 | 0, 1, or 2 | 0, 1, or 2 |
| Nuts*^*^* |  |  |  |
| Cereals | 0 to 1 | 0, 1, or 2 | 0, 1, or 2 |
| Dairy | 0 to 1 | 0, 1, or 2 | 0, 1, or 2 |
| Fish | 0 to 1 | 0, 1, or 2 | 0, 1, or 2 |
| Red meats^*^ | 0 to 1  0 to 1  0 to 1 | 0, 1, or 2 |  |
| Processed meats^*^ |  |  | 0, 1, or 2 |
| White meats^*^ |  |  |  |
| Eggs | 0 to 1 | - | - |
| Potatoes | 0 to 1 | - | - |
| Alcohol/Ethanol† | 0 to 1 | 0, 1, or 2 | 0, 1, or 2 |
| Sweets | 0 to 1 |  |  |
| Olive oil | 0 to 1 | 0, 1, or 2 | 0, 1, or 2 |

* In LitMDS and tMDS the following food groups were aggregated as a single component: 1) fruits and nuts, 2) red meat, processed meats, and white meats.

† In PyrMDS as alcohol beverages and in LitMDS and tMDS as ethanol (g/d).

**Table S2** Characteristics of participants included and excluded from the analysis, Fenland and CoLaus studies.

|  | Fenland study | | | | | | CoLaus study | | | | |
| --- | --- | --- | --- | --- | --- | --- | --- | --- | --- | --- | --- |
|  | Excluded  (n=2790) | FLI score | | Ultrasound liver fat score | |  | Excluded  (n=1107) | FLI score | | NAFLD Score | |
| Characteristic |  | Normal  (n=7035) | High  (n=2610) | Normal  (n=7342) | High  (n=2303) |  |  | Normal  (n=2955) | High  (n=1002) | Normal  (n=2939) | High  (n=1018) |
| Age, *years* | 47.4±7.6 | 48.4±7.5 | 50.3±7.2 | 48.5±7.5 | 50.1±7.1 |  | 60.5±10.6 | 56.4±10.3 | 58.7±10.5 | 56.1±10.3 | 59.5±10.3 |
| Women (%) | 51.3* | 62.6 | 32.8 | 58.5 | 41.9 |  | 41.5* | 64.2 | 35.0 | 62.5 | 40.5 |
| Marital status (%) † |  |  |  |  |  |  |  |  |  |  |  |
| Single | 6.2 | 7.9 | 5.6 | 7.7 | 5.9 |  | 13.7 | 16.0 | 13.7 | 16.4 | 12.6 |
| Married/cohabiting | 40.9 | 68.9 | 69.7 | 69.3 | 68.4 |  | 51.6 | 57.2 | 59.9 | 56.3 | 62.6 |
| Widowed/Separated/divorced | 4.8* | 8.3 | 7.4 | 8.1 | 7.8 |  | 30.4* | 26.8 | 26.4 | 27.4 | 24.9 |
| Occupation, (%) |  |  |  |  |  |  |  |  |  |  |  |
| Managerial and professional | 39.8* | 61.2 | 55.7 | 60.7 | 43.4 |  | - | - | - | - | - |
| Employed | - | - | - | - | - |  | 42.0* | 61.2 | 54.0 | 62.2 | 51.3 |
| Education (%) † |  |  |  |  |  |  |  |  |  |  |  |
| Compulsory | 24.5 | 17.3 | 21.5 | 17.7 | 20.8 |  | 25.6 | 13.9 | 18.5 | 13.7 | 18.8 |
| Secondary | 45.8 | 43.1 | 53.5 | 43.9 | 52.5 |  | 59.0 | 61.5 | 63.1 | 61.3 | 63.5 |
| University | 26.7* | 39.6 | 25.0 | 38.4 | 26.8 |  | 15.0* | 24.6 | 18.5 | 24.9 | 17.8 |
| Income, *per year* (%) † |  |  |  |  |  |  |  |  |  |  |  |
| <£20000 | 16.2 | 12.5 | 12.8 | 12.6 | 12.5 |  | - | - | - | - | - |
| £20000 – 40000 | 36.6 | 32.9 | 36.1 | 32.8 | 36.9 |  | - | - | - | - | - |
| >£40000 | 42.4* | 52.4 | 48.8 | 52.5 | 48.0 |  | - | - | - | - | - |
| Current smoker (%) | 15.3* | 10.8 | 12.9 | 11.2 | 11.8 |  | 23.6* | 21.3 | 20.0 | 21.9 | 18.0 |
| Alcohol consumption (unit/wk)†‡ |  |  |  |  |  |  |  |  |  |  |  |
| Abstainers | 5.9 | 3.8 | 4.1 | 3.9 | 3.9 |  | 32.1 | 23.8 | 22.4 | 23.5 | 23.0 |
| Moderate | 57.5 | 61.8 | 58.3 | 60.9 | 60.9 |  | 57.5 | 69.0 | 66.7 | 68.9 | 66.9 |
| Heavy | 24.9* | 25.6 | 29.5 | 26.5 | 27.1 |  | 10.4* | 7.2 | 11.0 | 7.5 | 10.1 |
| Total energy intake (kcal/d) | 2128±977* | 1901±562 | 1976±593 | 1916±567 | 1938±587 |  | 2039±1195* | 1783±601 | 1833±657 | 1782±609 | 1836±634 |
| Total protein (% energy) | 17.8±3.7* | 18.1±3.5 | 18.1±3.5 | 18.0±3.5 | 18.2±3.6 |  | 15.8±3.9* | 15.4 ±3.3 | 15.6±3.2 | 15.4±3.3 | 15.5±3.2 |
| Total Carbohydrate (% energy) | 48.3±7.0 | 48.5±6.9 | 47.2±7.1 | 48.4±6.9 | 47.5±7.1 |  | 45.2±9.5 | 46.7±8.6 | 45.3±9.2 | 46.6±8.7 | 45.7±8.9 |
| Total Fat (% energy) | 33.7±6.0 | 33.4±5.8 | 33.8±5.8 | 33.5±5.8 | 33.7±5.7 |  | 34.2±7.1 | 34.3±6.7 | 34.4±6.8 | 34.3±6.8 | 34.4±6.5 |
| PAEE (kcal/d) | 911±824 | 734±605 | 1082±881 | 776±657 | 993±829 |  | - | - | - | - | - |
| TEE (kcal/d) | - | - | - | - | - |  | 2761±690 | 2581±586 | 2935±673 | 2607±604 | 2846±660 |
| Metabolic syndrome (yes)§ | 44.3* | 17.3 | 75.1 | 23.7 | 62.3 |  | 55.6* | 19.1 | 72.7 | 13.7 | 87.2 |
| BMI (kg/m^2^) | 28.3±5.6* | 24.7±3.0 | 31.5±4.2 | 25.3±3.7 | 30.3±4.8 |  | 28.2±5.3* | 24.1±2.9 | 30.4±4.0 | 24.4±3.5 | 29.3±4.3 |
| Waist Circumference (cm) | 95.2±14.6* | 84.2±9.1 | 104.8±9.0 | 86.2±11.1 | 101.1±11.6 |  | 98.2±13.9* | 85.5±9.1 | 104.3±9.0 | 86.4±10.3 | 101.4±10.3 |
| Triglycerides (mmol/L) | 1.3±1.2* | 0.9±0.4 | 1.8±1.0 | 1.0±0.7 | 1.5±0.9 |  | 1.6±1.2* | 1.1±0.5 | 1.9±1.1 | 1.1±0.6 | 1.8±1.0 |
| median (iqr) | 1.1 (0.7, 1.6) | 0.8 (0.6, 1.1) | 1.6 (1.2, 2.2) | 0.9 (0.6, 1.2) | 1.3 (0.9, 1.9) |  | 1.3 (0.8, 1.9) | 1.0 (0.7, 1.3) | 1.7 (1.2, 2.2) | 1.0 (0.7, 1.3) | 1.5 (1.1, 2.1) |
| GGT (U/l) | 38.5±48.1* | 26.8±15.7 | 52.6±48.1 | 30.7±24.9 | 43.5 ± 42.8 |  | 49.3±59.9* | 25.4±20.8 | 60.0±72.6 | 26.6±21.4 | 55.9±73.2 |
| median (iqr) | 29 (21, 42) | 23 (18, 30) | 39 (29, 58) | 24 (19, 34) | 33 (25, 48) |  | 31 (21, 53) | 20 (15, 29) | 41 (28, 65) | 20 (15, 30) | 35 (25, 59) |
| ALT (U/l) | 31.7±18.7* | 24.4±12.2 | 38.3±19.7 | 26.0±14.0 | 35.0 ± 19.4 |  | 32.2±20.0* | 23.1±11.1 | 36.2±23.4 | 22.2±9.2 | 38.5±24.2 |
| median (iqr) | 27 (20, 38) | 22 (17, 29) | 34 (25, 46) | 23 (18, 31) | 30 (22, 43) |  | 27 (20, 38) | 21 (16, 27) | 30 (22, 43) | 20 (16, 26) | 32 (24, 44) |
| AST (U/l) | /- | - | - | - | - |  | 31.5±13.4* | 26.8±8.5 | 33.0±15.1 | 26.3±6.5 | 34.4±17.1 |
| median (iqr) | - | - | - | - | - |  | 28 (24, 35) | 25 (22, 30) | 30 (25, 36) | 25 (22, 29) | 30 (26, 37) |

Abbreviations: PAEE, physical activity energy expenditure; TEE, total energy expenditure; BMI, body mass index; iqr, interquartile range; GGT, gamma-glutamyl transferase; ALT, alanine aminotransferase; AST, aspartate aminotransferase.

Data are mean ± SD for continuous variables or percent for categorical variables, unless otherwise stated.

* Significant p-value (<0.05) for between-group comparisons of included and excluded participant using Chi-square test.

† Due to some missing data, percentages do not always add to 100%. ‘Separated’ included divorced or widowed adults.

‡ Alcohol consumption categorized as “abstainers” (0 unit/week), “moderate” (1–21 units/week for men, 1–14 for women), and “heavy drinkers” (>21 units/week for men, >14 for women).

§ Metabolic syndrome defined according to the International Diabetic Federation (see methods).

# **Table S3** Association between adherence to the literature-based and tertile-based Mediterranean diet and prevalence of hepatic steatosis, Fenland and CoLaus studies.

|  | Prevalence ratio (95% CI ) across quintiles of Mediterranean diet score* | | | | |  | Prevalence ratio  (95% CI)  Per SD difference* |
| --- | --- | --- | --- | --- | --- | --- | --- |
|  | Q1 | Q2 | Q3 | Q4 | Q5 | *P*-trend |  |
| Fenland Study |  |  |  |  |  |  |  |
| LitMDS, *range* | 2-8 | 9-10 | 11 | 12 | 13-18 |  |  |
| N Total | 2406 | 2798 | 1504 | 1284 | 1653 |  |  |
| Ultrasound liver fat score† |  |  |  |  |  |  |  |
| N Cases/total (score≥5 of 3 to 12) | 732 | 730 | 326 | 263 | 252 |  |  |
| Multivariable + SES + Dietary factor‡ | 1.00 (ref.) | 0.93 (0.82, 1.04) | 0.84 (0.72, 0.97) | 0.81 (0.68, 0.95) | 0.61 (0.51, 0.72) | <0.001 | 0.86 (0.82, 0.90) |
| Multivariable + BMI§ | 1.00 (ref.) | 1.01 (0.90, 1.14) | 0.93 (0.80, 1.08) | 0.94 (0.80, 1.11) | 0.80 (0.68, 0.95) | 0.009 | 0.94 (0.89, 0.99) |
| FLI, *median (iqr)*‖ | 45.8 (18.9, 74.9) | 34.5 (13.6, 65.2) | 29.0 (11.2, 58.5) | 24.3 (9.8, 50.8) | 18.2 (7.8, 44.0) |  |  |
| N cases (score>60) | 921 | 816 | 360 | 246 | 267 |  |  |
| Multivariable + SES + Dietary factor‡ | 1.00 (ref.) | 0.89 (0.80, 0.99) | 0.80 (0.69, 0.92) | 0.70 (0.59, 0.82) | 0.60 (0.51, 0.71) | <0.001 | 0.85 (0.82, 0.90) |
| Multivariable + BMI§ | 1.00 (ref.) | 1.02 (0.92, 1.14) | 0.94 (0.82, 1.08) | 0.87 (0.74, 1.02) | 0.87 (0.74, 1.03) | 0.02 | 0.96 (0.92, 1.01) |
|  |  |  |  |  |  |  |  |
| tMDS, *range* | 0-6 | 7-8 | 9-10 | 11-12 | 13-18 |  |  |
| N Total | 1,942 | 2,161 | 2,430 | 1,930 | 1,182 |  |  |
| Ultrasound liver fat score† |  |  |  |  |  |  |  |
| N Cases/total (score≥5 of 3 to 12) | 531 | 580 | 584 | 403 | 205 |  |  |
| Multivariable + SES + Dietary factor‡ | 1.00 (ref.) | 0.98 (0.86, 1.12) | 0.89 (0.78, 1.02) | 0.82 (0.70, 0.95) | 0.68 (0.57, 0.83) | <0.001 | 0.89 (0.85, 0.93) |
| Multivariable + BMI § | 1.00 (ref.) | 1.05 (0.92, 1.20) | 1.03 (0.90, 1.18) | 0.98 (0.85, 1.14) | 0.87 (0.72, 1.04) | 0.083 | 0.96 (0.92, 1.01) |
| FLI, *median (iqr)*‖ | 39.6 (16.1, 71.7) | 34.4 (12.6, 65.0) | 30.7 (11.2, 62.4) | 28.7 (11.0, 57.4) | 20.6 (9.0, 50.5) |  |  |
| N cases (score≥60) | 670 | 626 | 643 | 442 | 229 |  |  |
| Multivariable + SES + Dietary factor‡ | 1.00 (ref.) | 0.87 (0.77, 0.98) | 0.82 (0.72, 0.93) | 0.74 (0.64, 0.85) | 0.63 (0.53, 0.75) | <0.001 | 0.87 (0.83, 0.91) |
| Multivariable + BMI § | 1.00 (ref.) | 0.97 (0.86, 1.09) | 1.00 (0.89, 1.14) | 0.95 (0.83, 1.09) | 0.86 (0.72, 1.03) | 0.098 | 0.97 (0.93, 1.02) |
|  |  |  |  |  |  |  |  |
| CoLaus Study |  |  |  |  |  |  |  |
| LitMDS, *range* | 3-9 | 10-11 | 12 | 13 | 14-18 |  |  |
| N Total | 866 | 1,256 | 691 | 520 | 624 |  |  |
| FLI, *median (iqr)*‖ | 43.1 (17.5, 71.6) | 30.7 (13.2, 60.4) | 30.1 (13.2, 57.2) | 26.9 (10.8, 52.7) | 25.3 (10.9, 51.6) |  |  |
| N cases (score≥60) | 317 | 316 | 160 | 96 | 113 |  |  |
| Multivariable + SES + Dietary factor‡ | 1.00 (ref.) | 0.82 (0.69, 0.97) | 0.75 (0.61, 0.92) | 0.65 (0.51, 0.83) | 0.65 (0.51, 0.82) | <0.001 | 0.85 (0.80, 0.91) |
| Multivariable + BMI§ | 1.00 (ref.) | 0.91 (0.77, 1.08) | 0.95 (0.77, 1.17) | 0.83 (0.65, 1.07) | 0.79 (0.62, 1.01) | 0.045 | 0.93 (0.87, 1.002) |
| NAFLD liver fat score, *median (iqr)* ** | -1.5 (-2.3, -0.3) | -1.8 (-2.4, -0.65) | -1.7 (-2.4, -0.6) | -1.9 (-2.5, -0.9) | -1.8 (-2.4, -0.8) |  |  |
| N cases | 272 | 312 | 182 | 115 | 137 |  |  |
| Multivariable + SES + Dietary factor‡ | 1.00 (ref.) | 0.89 (0.75, 1.06) | 0.94 (0.77, 1.15) | 0.83 (0.66, 1.05) | 0.84 (0.67, 1.05) | 0.096 | 0.93 (0.87, 0.99) |
| Multivariable + BMI§ | 1.00 (ref.) | 0.96 (0.80, 1.14) | 1.11 (0.91, 1.37) | 1.01 (0.80, 1.28) | 0.97 (0.77, 1.22) | 0.96 | 0.99 (0.93, 1.06) |
|  |  |  |  |  |  |  |  |
| tMDS, *range* | 0-7 | 8-9 | 10 | 11 | 12-18 |  |  |
| N Total | 956 | 1777 | 562 | 500 | 762 |  |  |
| FLI, *median (iqr)*‖ | 36.6 (13.8, 67.0) | 29.7 (13.2, 59.2) | 32.4 (13.0, 61.8) | 31.8 (14.1, 57.3) | 29.1 (11.8, 54.4) |  |  |
| N cases (score≥60) | 295 | 289 | 144 | 112 | 162 |  |  |
| Multivariable + SES + Dietary factor‡ | 1.00 (ref.) | 0.88 (0.73, 1.04) | 0.86 (0.69, 1.06) | 0.76 (0.60, 0.96) | 0.73 (0.59, 0.89) | 0.001 | 0.89 (0.83, 0.95) |
| Multivariable + BMI§ | 1.00 (ref.) | 0.93 (0.78, 1.12) | 1.01 (0.81, 1.26) | 0.95 (0.75, 1.21) | 0.88 (0.72, 1.09) | 0.35 | 0.96 (0.90, 1.03) |
| NAFLD liver fat score, *median (iqr)* ** | -1.6 (-2.3, -0.4) | -1.7 (-2.4, -0.6) | -1.8 (-2.4, -0.6) | -1.8 (-2.4, -0.7) | -1.9 (-2.4, -0.7) |  |  |
| N cases (score≥-0.640) | 268 | 302 | 151 | 119 | 178 |  |  |
| Multivariable + SES + Dietary factor‡ | 1.00 (ref.) | 0.99 (0.83, 1.18) | 1.01 (0.81, 1.25) | 0.88 (0.70, 1.11) | 0.89 (0.72, 1.09) | 0.13 | 0.94 (0.88, 1.01) |
| Multivariable + BMI§ | 1.00 (ref.) | 1.03 (0.86, 1.23) | 1.12 (0.90, 1.39) | 1.04 (0.82, 1.31) | 1.02 (0.83, 1.25) | 0.84 | 0.99 (0.93, 1.06) |

Abbreviations: FLI, fatty liver index; iqr, interquartile range; SES, socio economic status; BMI, body mass index; NAFLD, non-alcoholic fatty liver disease.

* In categorical analysis, the population was divided into five groups by quintiles (Q1-Q5) of the Mediterranean diet score, Standard deviation is 2.4 and 2.24 for literature-based Mediterranean diet score, and 2.93 and 2.53 for tertile-based Mediterranean diet score in the Fenland and CoLaus study, respectively.

† Assessed by abdominal ultrasound.

‡ Adjusted for age (years), sex, marital status (single, married/cohabitant, and divorced/widowed), occupational status (routine and professional jobs in the Fenland study and working and not working in the CoLaus study), education level (compulsory, secondary, and university), smoking status (never, former, and current), energy intake (kcal/day), physical activity energy expenditure (kcal/d, in the Fenland study), total energy expenditure (kcal/day, in the CoLaus study), and date of dietary assessment (in the CoLaus study).

§ Further adjusted for BMI. Results of further adjustment for waist circumference were broadly in line with of the further adjustment for BMI (data not shown).

‖ Calculated based on an algorithm including body mass index, waist circumference, triglycerides, and gamma-glutamyl transferase.

** Calculated based on an algorithm including presence of the metabolic syndrome and type 2 diabetes, and concentrations of fasting serum insulin, fasting serum aspartate-aminotransferase (AST), and the AST/alanine-aminotransferase ratio.

# **Table S4** Association between adherence to the Mediterranean diet and prevalence of hepatic steatosis within alcohol consumption strata, Fenland and CoLaus studies.

|  |  | Prevalence ratio (95% CI) across quintiles of pyramid-based Mediterranean diet score* | | | | | *P*-trend | Prevalence ratio (95% CI)  per SD difference* |
| --- | --- | --- | --- | --- | --- | --- | --- | --- |
|  |  | Q1 | Q2 | Q3 | Q4 | Q5 |  |  |
| Fenland Study, *range* | | 3.30-7.84 | 7.85-8.73 | 8.74-9.46 | 9.47-10.28 | 10.29-14.03 |  |  |
| N Total | | 1929 | 1929 | 1929 | 1929 | 1929 |  |  |
|  | Ultrasound liver fat score† |  |  |  |  |  |  |  |
| Abstainers | N Cases/total (score≥5 of 3 to 12) | 26/79 | 26/82 | 16/86 | 15/76 | 7/50 |  |  |
|  | Multivariable + SES + Dietary factor‡ | 1.00 (ref.) | 0.88 (0.44, 1.80) | 0.59 (0.29, 1.22) | 0.64 (0.29, 1.41) | 0.43 (0.15, 1.21) | 0.08 | 0.76 (0.58, 1.00) |
|  | Multivariable + BMI§ | 1.00 (ref.) | 0.68 (0.32, 1.43) | 0.65 (0.31, 1.37) | 0.74 (0.33, 1.66) | 0.71 (0.25, 2.03) | 0.61 | 0.89 (0.66, 1.20) |
| Moderate drinkers | N Cases/total (score≥5 of 3 to 12) | 363/1,168 | 340/1189 | 261/1171 | 247/1173 | 191/1170 |  |  |
|  | Multivariable + SES + Dietary factor‡ | 1.00 (ref.) | 1.01 (0.86, 1.19) | 0.80 (0.67, 0.97) | 0.81 (0.67, 0.99) | 0.72 (0.58, 0.88) | <0.001 | 0.87 (0.81, 0.93) |
|  | Multivariable + BMI§ | 1.00 (ref.) | 1.03 (0.87, 1.22) | 0.92 (0.00,1.10) | 0.96 (0.79, 1.16) | 0.92 (0.75, 1.14) | 0.33 | 0.96 (0.90, 1.03) |
| Heavy drinkers | N Cases/total (score≥5 of 3 to 12) | 145/438 | 147/499 | 137/530 | 108/535 | 87/570 |  |  |
|  | Multivariable + SES + Dietary factor‡ | 1.00 (ref.) | 0.98 (0.75, 1.28) | 0.91 (0.69, 1.21) | 0.70 (0.52, 0.94) | 0.56 (0.40, 0.77) | <0.001 | 0.82 (0.75, 0.91) |
|  | Multivariable + BMI§ | 1.00 (ref.) | 1.07 (0.82, 1.40) | 1.02 (0.77, 1.36) | 0.78 (0.58, 1.05) | 0.73 (0.52, 1.02) | 0.011 | 0.91 (0.82, 1.00) |
|  |  |  |  |  |  |  |  |  |
|  | FLI‖ |  |  |  |  |  |  |  |
| Abstainers | N Cases/total (score≥60) | 29/79 | 30/82 | 22/86 | 19/76 | 8/50 |  |  |
|  | Multivariable + SES + Dietary factor‡ | 1.00 (ref.) | 1.24 (0.66, 2.36) | 0.78 (0.39, 1.52) | 1.03 (0.51, 2.08) | 0.50 (0.18, 1.38) | 0.15 | 0.85 (0.66, 1.09) |
|  | Multivariable + BMI§ | 1.00 (ref.) | 1.15 (0.59, 2.26) | 0.98 (0.48, 1.99) | 1.35 (0.64, 2.82) | 0.92 (0.33, 2.60) | 0.99 | 1.04 (0.79, 1.37) |
| Moderate drinkers | N Cases/total (score≥60) | 443/1,168 | 373/1,189 | 286/1,171 | 251/1,173 | 168/1,170 |  |  |
|  | Multivariable + SES + Dietary factor‡ | 1.00 (ref.) | 0.96 (0.83, 1.12) | 0.83 (0.70, 0.98) | 0.77 (0.64, 0.93) | 0.56 (0.46, 0.70) | <0.001 | 0.83 (0.78, 0.88) |
|  | Multivariable + BMI§ | 1.00 (ref.) | 1.01 (0.86, 1.17) | 1.02 (0.87, 1.21) | 0.99 (0.82, 1.18) | 0.80 (0.65, 0.98) | 0.045 | 0.95 (0.89, 1.01) |
| Heavy drinkers | N Cases/total (score≥60) | 206/438 | 185/499 | 153/530 | 134/535 | 92/570 |  |  |
|  | Multivariable + SES + Dietary factor‡ | 1.00 (ref.) | 0.94 (0.75, 1.19) | 0.84 (0.65, 1.08) | 0.77 (0.60, 1.00) | 0.53 (0.39, 0.72) | <0.001 | 0.83 (0.76, 0.91) |
|  | Multivariable + BMI§ | 1.00 (ref.) | 1.04 (0.82, 1.32) | 0.99 (0.76, 1.28) | 0.89 (0.69, 1.15) | 0.77 (0.57, 1.05) | 0.051 | 0.94 (0.86, 1.03) |
|  |  |  |  |  |  |  |  |  |
| CoLaus Study, *range* | | 1.83-7.45 | 7.46-8.18 | 8.19-8.82 | 8.83-9.47 | 9.48-12.18 |  |  |
| N Total | | 792 | 791 | 792 | 791 | 791 |  |  |
|  | FLI‖ |  |  |  |  |  |  |  |
| Abstainers | N Cases/total (score≥60) | 52/167 | 54/184 | 40/190 | 42/194 | 36/191 |  |  |
|  | Multivariable + SES + Dietary factor‡ | 1.00 (ref.) | 1.06 (0.68, 1.66) | 0.84 (0.53, 1.33) | 0.94 (0.59, 1.49) | 0.8 (0.49, 1.30) | 0.28 | 0.93 (0.80, 1.08) |
|  | Multivariable + BMI§ | 1.00 (ref.) | 0.93 (0.58, 1.48) | 0.94 (0.59, 1.50) | 0.98 (0.61, 1.57) | 0.8 (0.49, 1.32) | 0.49 | 0.94 (0.81, 1.09) |
| Moderate drinkers | N Cases/total (score≥60) | 186/495 | 164/521 | 115/554 | 110/560 | 93/577 |  |  |
|  | Multivariable + SES + Dietary factor‡ | 1.00 (ref.) | 0.97 (0.77, 1.21) | 0.75 (0.58, 0.96) | 0.73 (0.57, 0.94) | 0.58 (0.44, 0.77) | <0.001 | 0.83 (0.77, 0.91) |
|  | Multivariable + BMI§ | 1.00 (ref.) | 1.07 (0.85, 1.35) | 0.84 (0.65, 1.08) | 0.82 (0.64, 1.06) | 0.79 (0.60, 1.05) | 0.022 | 0.92 (0.84, 1.00) |
| Heavy drinkers | N Cases/total (score≥60) | 59/130 | 22/86 | 15/48 | 8/37 | 6/23 |  |  |
|  | Multivariable + SES + Dietary factor‡ | 1.00 (ref.) | 0.65 (0.38, 1.10) | 0.72 (0.38, 1.35) | 0.52 (0.23, 1.19) | 0.75 (0.32, 1.78) | 0.43 | 0.89 (0.72, 1.09) |
|  | Multivariable + BMI§ | 1.00 (ref.) | 0.75 (0.43, 1.33) | 0.95 (0.50, 1.81) | 0.75 (0.33, 1.73) | 0.91 (0.38, 2.19) | 0.85 | 0.94 (0.75, 1.17) |
|  |  |  |  |  |  |  |  |  |
|  | NAFLD liver fat score** |  |  |  |  |  |  |  |
| Abstainers | N Cases/total (score≥-0.640) | 56/167 | 52/184 | 38/190 | 44/194 | 44/191 |  |  |
|  | Multivariable + SES + Dietary factor‡ | 1.00 (ref.) | 0.95 (0.62, 1.46) | 0.68 (0.43, 1.07) | 0.81 (0.51, 1.27) | 0.90 (0.57, 1.41) | 0.45 | 0.94 (0.81, 1.09) |
|  | Multivariable + BMI§ | 1.00 (ref.) | 0.86 (0.55, 1.33) | 0.72 (0.46, 1.14) | 0.80 (0.51, 1.26) | 0.91 (0.58, 1.43) | 0.60 | 0.94 (0.81, 1.09) |
| Moderate drinkers | N Cases/total (score≥-0.640) | 150/495 | 164/521 | 131/554 | 117/560 | 119/577 |  |  |
|  | Multivariable + SES + Dietary factor‡ | 1.00 (ref.) | 1.24 (0.97, 1.57) | 0.96 (0.75, 1.23) | 0.91 (0.70, 1.17) | 0.86 (0.66, 1.13) | 0.077 | 0.93 (0.86, 1.01) |
|  | Multivariable + BMI§ | 1.00 (ref.) | 1.24 (0.97, 1.57) | 1.06 (0.00, 1.37) | 0.99 (0.77, 1.28) | 1.10 (0.84, 1.44) | 0.93 | 1.01 (0.92, 1.10) |
| Heavy drinkers | N Cases/total (score≥-0.640) | 51/130 | 24/86 | 12/48 | 7/37 | 9/23 |  |  |
|  | Multivariable + SES + Dietary factor‡ | 1.00 (ref.) | 0.74 (0.44, 1.27) | 0.67 (0.33, 1.34) | 0.48 (0.20, 1.17) | 1.19 (0.57, 2.47) | 0.92 | 0.96 (0.77, 1.19) |
|  | Multivariable + BMI§ | 1.00 (ref.) | 0.83 (0.47, 1.45) | 0.80 (0.40, 1.61) | 0.62 (0.26, 1.51) | 1.37 (0.65, 2.90) | 0.71 | 1.00 (0.80, 1.26) |

Abbreviations: FLI, fatty liver index; SES, socio economic status; BMI, body mass index; NAFLD, non-alcoholic fatty liver disease; NE, not estimated.

Alcohol consumption categorized as “abstainers” (0 unit/week), “moderate” (1–21 units/week for men, 1–14 for women), and “heavy drinkers” (>21 units/week for men, >14 for women).

* In categorical analysis, the population was divided into five groups by quintiles (Q1-Q5) of the Mediterranean diet score, Standard deviation is 1.43 and 1.24 for pyramid-based Mediterranean diet score, in the Fenland and CoLaus studies, respectively.

† Assessed by abdominal ultrasound.

‡ Adjusted for age (years), sex, marital status (single, married/cohabitant, and divorced/widowed), occupational status (routine and professional jobs in the Fenland study and working and not working in the CoLaus study), education level (compulsory, secondary, and university), smoking status (never, former, and current), energy intake (kcal/day), physical activity energy expenditure (kcal/d, in the Fenland study), total energy expenditure (kcal/day, in the CoLaus study), and date of dietary assessment (in the CoLaus study).

§ Further adjusted for BMI.

‖ Calculated based on an algorithm including body mass index, waist circumference, triglycerides, and gamma-glutamyl transferase.

** Calculated based on an algorithm including presence of the metabolic syndrome and type 2 diabetes, and concentrations of fasting serum insulin, fasting serum aspartate-aminotransferase (AST), and the AST/alanine-aminotransferase ratio.

# **Table S5** Sensitivity analyses for the association between adherence to the Mediterranean diet and prevalence of hepatic steatosis, Fenland and CoLaus studies.

|  | Prevalence ratio (95% CI) across quintiles of pyramid-based Mediterranean diet score* | | | | | *P*-trend | Prevalence ratio  (95% CI)  Per SD difference* |
| --- | --- | --- | --- | --- | --- | --- | --- |
|  | Q1 | Q2 | Q3 | Q4 | Q5 |  |  |
| Fenland Study, *range* | 3.30-7.84 | 7.85-8.73 | 8.74-9.46 | 9.47-10.28 | 10.29-14.03 |  |  |
| N total | 1929 | 1929 | 1929 | 1929 | 1929 |  |  |
| Ultrasound liver fat score |  |  |  |  |  |  |  |
| Different models |  |  |  |  |  |  |  |
| N Cases (score≥5 of 3 to 12) | 602 | 547 | 450 | 391 | 313 |  |  |
| Multivariable + SES + Dietary factor (Model 1)† | 1.00 (ref.) | 0.99 (0.86, 1.12) | 0.82 (0.71, 0.95) | 0.76 (0.65, 0.88) | 0.67 (0.56, 0.78) | <0.001 | 0.86 (0.81, 0.90) |
| Model 1 – Marital status | 1.00 (ref.) | 0.96 (0.86, 1.09) | 0.84 (0.74, 0.95) | 0.77 (0.67, 0.88) | 0.66 (0.56, 0.76) | <0.001 | 0.86 (0.82, 0.90) |
| Model 1 + BMI & WC | 1.00 (ref.) | 1.05 (0.92, 1.20) | 0.99 (0.85, 1.14) | 0.95 (0.82, 1.11) | 0.95 (0.81, 1.13) | 0.31 | 0.98 (0.93, 1.03) |
| Model 1 + Body fat mass | 1.00 (ref.) | 0.99 (0.86, 1.14) | 0.82 (0.71, 0.96) | 0.77 (0.66, 0.90) | 0.65 (0.55, 0.77) | <0.001 | 0.85 (0.81, 0.90) |
| Model 1 + Alcohol | 1.00 (ref.) | 1.06 (0.93, 1.22) | 0.99 (0.86, 1.15) | 0.95 (0.81, 1.12) | 0.93 (0.78, 1.11) | 0.19 | 0.97 (0.92, 1.03) |
| Model 1 + Alcohol+ BMI & WC | 1.00 (ref.) | 1.00 (0.88, 1.14) | 0.96 (0.83, 1.11) | 0.90 (0.78, 1.05) | 0.92 (0.78, 1.09) | 0.17 | 0.96 (0.91, 1.02) |
| Model 1 + Clinical variables‡ | 1.00 (ref.) | 1.02 (0.89, 1.16) | 0.86 (0.75, 1.00) | 0.82 (0.70, 0.95) | 0.73 (0.62, 0.87) | <0.001 | 0.89 (0.84, 0.94) |
| Model 1 + Clinical variables + BMI & WC | 1.00 (ref.) | 1.06 (0.93, 1.22) | 0.98 (0.85, 1.14) | 0.97 (0.83, 1.13) | 0.97 (0.82, 1.15) | 0.40 | 0.98 (0.93, 1.04) |
| Model 1 + Family history of T2D + MetS§ | 1.00 (ref.) | 1.03 (0.90, 1.17) | 0.88 (0.76, 1.02) | 0.81 (0.70, 0.95) | 0.78 (0.66, 0.92) | <0.001 | 0.90 (0.86, 0.95) |
| Excluding alcohol from MD component |  |  |  |  |  |  |  |
| Model 1 + Alcohol | 1.00 (ref.) | 0.97 (0.85, 1.12) | 0.85 (0.73, 0.99) | 0.77 (0.66, 0.90) | 0.63 (0.53, 0.75) | <0.001 | 0.85 (0.81, 0.90) |
| Model 1 + Alcohol + BMI | 1.00 (ref.) | 1.09 (0.95, 1.25) | 1.02 (0.88, 1.19) | 0.98 (0.84, 1.15) | 0.94 (0.79, 1.12) | 0.25 | 0.97 (0.92, 1.03) |
| Excluding participants with BMI≥30‖ |  |  |  |  |  |  |  |
| Model 1 | 1.00 (ref.) | 0.95 (0.79, 1.14) | 0.81 (0.67, 0.99) | 0.83 (0.68, 1.02) | 0.74 (0.59, 0.92) | 0.003 | 0.90 (0.84, 0.96) |
| Model 1 + BMI | 1.00 (ref.) | 0.95 (0.79, 1.13) | 0.86 (0.70, 1.04) | 0.88 (0.72, 1.08) | 0.89 (0.72, 1.11) | 0.23 | 0.95 (0.88, 1.02) |
| Excluding participants with excessive alcohol consumption** |  |  |  |  |  |  |  |
| Model 1 | 1.00 (ref.) | 1.00 (0.87, 1.15) | 0.82 (0.70, 0.96) | 0.78 (0.66, 0.92) | 0.70 (0.58, 0.84) | <0.001 | 0.87 (0.82, 0.92) |
| Model 1 + BMI | 1.00 (ref.) | 1.03 (0.89, 1.18) | 0.94 (0.81, 1.10) | 0.90 (0.77, 1.07) | 0.91 (0.76, 1.09) | 0.13 | 0.96 (0.90, 1.01) |
| Including participants with probable implausible energy intake†† |  |  |  |  |  |  |  |
| Model 1 | 1.00 (ref.) | 0.99 (0.87, 1.12) | 0.82 (0.71, 0.95) | 0.78 (0.67, 0.91) | 0.67 (0.57, 0.79) | <0.001 | 0.86 (0.82, 0.91) |
| Model 1 + BMI | 1.00 (ref.) | 1.01 (0.89, 1.15) | 0.94 (0.82, 1.09) | 0.92 (0.79, 1.07) | 0.89 (0.75, 1.04) | 0.07 | 0.95 (0.91, 1.003) |
| Excluding participants with secondary causes of hepatic steatosis‡‡ |  |  |  |  |  |  |  |
| Model 1 | 1.00 (ref.) | 0.99 (0.86, 1.13) | 0.83 (0.72, 0.96) | 0.75 (0.64, 0.87) | 0.66 (0.56, 0.78) | <0.001 | 0.85 (0.81, 0.90) |
| Model 1 + BMI | 1.00 (ref.) | 1.02 (0.89, 1.16) | 0.95 (0.82, 1.09) | 0.90 (0.77, 1.05) | 0.87 (0.74, 1.03) | 0.035 | 0.95 (0.90, 0.99) |
|  |  |  |  |  |  |  |  |
| FLI§§ |  |  |  |  |  |  |  |
| Different models |  |  |  |  |  |  |  |
| N Cases/total (score≥60) | 773 | 632 | 496 | 424 | 285 |  |  |
| Multivariable + SES + Dietary factor (Model 1)† | 1.00 (ref.) | 0.94 (0.84, 1.06) | 0.81 (0.71, 0.92) | 0.75 (0.65, 0.86) | 0.52 (0.44, 0.62) | <0.001 | 0.82 (0.78, 0.86) |
| Model 1 – Marital status | 1.00 (ref.) | 0.94 (0.84, 1.04) | 0.82 (0.73, 0.92) | 0.76 (0.67, 0.86) | 0.56 (0.48, 0.65) | <0.001 | 0.83 (0.80, 0.87) |
| Model 1 + BMI & WC | 1.00 (ref.) | 1.04 (0.92, 1.17) | 1.04 (0.91, 1.18) | 1.02 (0.89, 1.17) | 0.83 (0.70, 0.98) | 0.036 | 0.97 (0.93, 1.02) |
| Model 1 + Alcohol | 1.00 (ref.) | 0.96 (0.85, 1.08) | 0.99 (0.87, 1.13) | 0.93 (0.81, 1.07) | 0.80 (0.68, 0.95) | 0.011 | 0.95 (0.91, 1.001) |
| Model 1 + Alcohol+ BMI & WC | 1.00 (ref.) | 0.96 (0.85, 1.09) | 0.83 (0.73, 0.96) | 0.79 (0.68, 0.91) | 0.55 (0.47, 0.65) | <0.001 | 0.83 (0.79, 0.87) |
| Model 1 + Body fat mass | 1.00 (ref.) | 1.07 (0.94, 1.21) | 1.08 (0.94, 1.24) | 1.06 (0.91, 1.22) | 0.87 (0.73, 1.03) | 0.12 | 0.99 (0.94, 1.04) |
| Model 1 + Clinical variables‡ | 1.00 (ref.) | 0.98 (0.87, 1.11) | 0.89 (0.77, 1.01) | 0.84 (0.73, 0.96) | 0.62 (0.52, 0.73) | <0.001 | 0.87 (0.82, 0.91) |
| Model 1 + Clinical variables + BMI & WC | 1.00 (ref.) | 1.05 (0.93, 1.19) | 1.06 (0.92, 1.21) | 1.04 (0.91, 1.20) | 0.86 (0.73, 1.02) | 0.10 | 0.98 (0.93, 1.03) |
| Model 1 + Family history of T2D + MetS§ | 1.00 (ref.) | 1.00 (0.88, 1.12) | 0.91 (0.80, 1.04) | 0.83 (0.73, 0.96) | 0.67 (0.57, 0.79) | <0.001 | 0.89 (0.84, 0.93) |
| Excluding alcohol from MD component |  |  |  |  |  |  |  |
| Model 1 + Alcohol | 1.00 (ref.) | 0.97 (0.85, 1.10) | 0.84 (0.73, 0.96) | 0.78 (0.68, 0.91) | 0.52 (0.43, 0.61) | <0.001 | 0.82 (0.78, 0.87) |
| Model 1 + Alcohol + BMI | 1.00 (ref.) | 1.14 (1.00, 1.29) | 1.07 (0.93, 1.22) | 1.09 (0.94, 1.26) | 0.87 (0.73, 1.04) | 0.10 | 0.98 (0.93, 1.03) |
| Excluding participants with BMI≥30‖ |  |  |  |  |  |  |  |
| Model 1 | 1.00 (ref.) | 0.95 (0.80, 1.14) | 0.83 (0.68, 1.01) | 0.71 (0.57, 0.88) | 0.50 (0.38, 0.65) | <0.001 | 0.81 (0.75, 0.88) |
| Model 1 + BMI | 1.00 (ref.) | 0.97 (0.81, 1.16) | 0.89 (0.73, 1.08) | 0.80 (0.64, 0.99) | 0.69 (0.53, 0.90) | 0.001 | 0.88 (0.81, 0.95) |
| Excluding participants with excessive alcohol consumption** |  |  |  |  |  |  |  |
| Model 1 | 1.00 (ref.) | 0.96 (0.85, 1.09) | 0.82 (0.72, 0.95) | 0.79 (0.68, 0.92) | 0.56 (0.47, 0.67) | <0.001 | 0.83 (0.79, 0.88) |
| Model 1 + BMI | 1.00 (ref.) | 1.01 (0.89, 1.15) | 1.00 (0.87, 1.15) | 0.98 (0.84, 1.13) | 0.80 (0.67, 0.96) | 0.015 | 0.95 (0.90, 1.004) |
| Including participants with probable implausible energy intake†† |  |  |  |  |  |  |  |
| Model 1 | 1.00 (ref.) | 0.95 (0.84, 1.07) | 0.81 (0.72, 0.93) | 0.76 (0.66, 0.87) | 0.53 (0.45, 0.62) | <0.001 | 0.82 (0.78, 0.86) |
| Model 1 + BMI | 1.00 (ref.) | 0.99 (0.88, 1.12) | 0.99 (0.87, 1.13) | 0.95 (0.83, 1.09) | 0.76 (0.65, 0.90) | 0.001 | 0.94 (0.89, 0.98) |
| Excluding participants with secondary causes of hepatic steatosis‡‡ |  |  |  |  |  |  |  |
| Model 1 | 1.00 (ref.) | 0.94 (0.83, 1.06) | 0.80 (0.70, 0.92) | 0.73 (0.64, 0.85) | 0.52 (0.44, 0.61) | <0.001 | 0.81 (0.77, 0.85) |
| Model 1 + BMI | 1.00 (ref.) | 0.99 (0.88, 1.12) | 0.98 (0.86, 1.12) | 0.94 (0.82, 1.09) | 0.75 (0.64, 0.89) | 0.001 | 0.94 (0.89, 0.98) |
|  |  |  |  |  |  |  |  |
| CoLaus Study, *range* | 1.83-7.45 | 7.46-8.18 | 8.19-8.82 | 8.83-9.47 | 9.48-12.18 |  |  |
| N total | 792 | 791 | 792 | 791 | 791 |  |  |
| FLI§§ |  |  |  |  |  |  |  |
| Different models |  |  |  |  |  |  |  |
| N Cases (score≥60) | 297 | 240 | 170 | 160 | 135 |  |  |
| Multivariable + SES + Dietary factor (Model 1)† | 1.00 (ref.) | 0.93 (0.77, 1.12) | 0.74 (0.60, 0.90) | 0.73 (0.59, 0.90) | 0.61 (0.49, 0.77) | <0.001 | 0.85 (0.80, 0.91) |
| Model 1 – Marital status | 1.00 (ref.) | 0.93 (0.77, 1.12) | 0.74 (0.60, 0.90) | 0.73 (0.59, 0.90) | 0.61 (0.49, 0.77) | <0.001 | 0.85 (0.80, 0.91) |
| Model 1 + BMI & WC | 1.00 (ref.) | 1.06 (0.87, 1.28) | 0.90 (0.74, 1.11) | 0.85 (0.69, 1.05) | 0.79 (0.63, 1.00) | 0.009 | 0.93 (0.87, 0.99) |
| Model 1 + Alcohol | 1.00 (ref.) | 0.93 (0.78, 1.13) | 0.75 (0.61, 0.91) | 0.74 (0.60, 0.91) | 0.62 (0.50, 0.78) | <0.001 | 0.86 (0.80, 0.92) |
| Model 1 + Alcohol+ BMI & WC | 1.00 (ref.) | 1.07 (0.89, 1.30) | 0.93 (0.75, 1.14) | 0.88 (0.71, 1.09) | 0.82 (0.65, 1.03) | 0.023 | 0.95 (0.88, 1.02) |
| Model 1 – Date of assessment | 1.00 (ref.) | 0.93 (0.77, 1.12) | 0.74 (0.60, 0.90) | 0.73 (0.59, 0.90) | 0.62 (0.49, 0.77) | <0.001 | 0.85 (0.80, 0.91) |
| Model 1 + Clinical variables‡ | 1.00 (ref.) | 0.87 (0.72, 1.06) | 0.80 (0.65, 0.99) | 0.81 (0.65, 1.01) | 0.66 (0.52, 0.84) | 0.001 | 0.88 (0.82, 0.95) |
| Model 1 + Clinical variables + BMI & WC | 1.00 (ref.) | 0.99 (0.81, 1.21) | 0.90 (0.73, 1.12) | 0.90 (0.72, 1.12) | 0.78 (0.61, 1.00) | 0.033 | 0.93 (0.86, 1.002) |
| Model 1 + Family history of T2D + MetS§ | 1.00 (ref.) | 0.88 (0.73, 1.08) | 0.76 (0.61, 0.93) | 0.78 (0.63, 0.97) | 0.64 (0.51, 0.82) | <0.001 | 0.88 (0.82, 0.94) |
| Excluding alcohol from MD component |  |  |  |  |  |  |  |
| Model 1 + Alcohol | 1.00 (ref.) | 1.00 (0.83, 1.20) | 0.72 (0.59, 0.89) | 0.78 (0.63, 0.96) | 0.62 (0.50, 0.78) | <0.001 | 0.85 (0.79, 0.91) |
| Model 1 + Alcohol + BMI | 1.00 (ref.) | 1.12 (0.93, 1.36) | 0.83 (0.68, 1.02) | 0.82 (0.67, 1.01) | 0.80 (0.63, 1.00) | 0.003 | 0.90 (0.84, 0.96) |
| Excluding participants with BMI≥30‖ |  |  |  |  |  |  |  |
| Model 1 | 1.00 (ref.) | 1.01 (0.79, 1.30) | 0.75 (0.56, 0.99) | 0.64 (0.47, 0.86) | 0.64 (0.47, 0.88) | <0.001 | 0.84 (0.77, 0.92) |
| Model 1 + BMI | 1.00 (ref.) | 0.98 (0.76, 1.25) | 0.76 (0.58, 1.01) | 0.64 (0.47, 0.86) | 0.77 (0.56, 1.05) | 0.007 | 0.87 (0.79, 0.95) |
| Excluding participants with excessive alcohol consumption** |  |  |  |  |  |  |  |
| Model 1 | 1.00 (ref.) | 0.95 (0.78, 1.15) | 0.75 (0.61, 0.93) | 0.74 (0.60, 0.92) | 0.62 (0.49, 0.78) | <0.001 | 0.86 (0.80, 0.92) |
| Model 1 + BMI | 1.00 (ref.) | 1.01 (0.83, 1.22) | 0.83 (0.67, 1.02) | 0.83 (0.67, 1.03) | 0.78 (0.62, 0.99) | 0.01 | 0.92 (0.85, 0.98) |
| Including participants with probable implausible energy intake†† |  |  |  |  |  |  |  |
| Model 1 | 1.00 (ref.) | 0.92 (0.76, 1.10) | 0.73 (0.60, 0.89) | 0.72 (0.59, 0.88) | 0.62 (0.50, 0.77) | <0.001 | 0.85 (0.79, 0.91) |
| Model 1 + BMI | 1.00 (ref.) | 0.98 (0.82, 1.19) | 0.82 (0.67, 1.00) | 0.81 (0.66, 1.00) | 0.74 (0.59, 0.92) | 0.001 | 0.90 (0.85, 0.97) |
| Excluding participants with secondary causes of hepatic steatosis‡‡ |  |  |  |  |  |  |  |
| Model 1 | 1.00 (ref.) | 0.91 (0.76, 1.10) | 0.74 (0.60, 0.90) | 0.72 (0.58, 0.88) | 0.61 (0.49, 0.77) | <0.001 | 0.85 (0.79, 0.91) |
| Model 1 + BMI | 1.00 (ref.) | 0.99 (0.82, 1.20) | 0.82 (0.67, 1.01) | 0.81 (0.65, 1.00) | 0.77 (0.61, 0.97) | 0.006 | 0.91 (0.85, 0.98) |
|  |  |  |  |  |  |  |  |
| NAFLD liver fat score†† |  |  |  |  |  |  |  |
| Different models |  |  |  |  |  |  |  |
| N Cases (score≥-0.640) | 257 | 240 | 181 | 168 | 172 |  |  |
| Multivariable + SES + Dietary factor (Model 1)† | 1.00 (ref.) | 1.03 (0.86, 1.25) | 0.84 (0.69, 1.03) | 0.82 (0.67, 1.01) | 0.85 (0.69, 1.05) | 0.022 | 0.93 (0.87, 0.99) |
| Model 1 – Marital status | 1.00 (ref.) | 1.03 (0.86, 1.25) | 0.84 (0.69, 1.03) | 0.82 (0.67, 1.01) | 0.85 (0.69, 1.05) | 0.022 | 0.93 (0.87, 0.99) |
| Model 1 + BMI & WC | 1.00 (ref.) | 1.12 (0.92, 1.36) | 0.98 (0.80, 1.20) | 0.92 (0.74, 1.13) | 1.05 (0.85, 1.30) | 0.69 | 0.99 (0.93, 1.06) |
| Model 1 + Alcohol | 1.00 (ref.) | 1.04 (0.86, 1.26) | 0.85 (0.69, 1.05) | 0.83 (0.68, 1.03) | 0.87 (0.70, 1.07) | 0.039 | 0.93 (0.87, 1.001) |
| Model 1 + Alcohol+ BMI & WC | 1.00 (ref.) | 1.13 (0.93, 1.37) | 1.00 (0.82, 1.24) | 0.94 (0.76, 1.17) | 1.08 (0.87, 1.35) | 0.91 | 1.01 (0.94, 1.08) |
| Model 1 – Date of assessment | 1.00 (ref.) | 1.04 (0.86, 1.25) | 0.84 (0.69, 1.03) | 0.82 (0.67, 1.01) | 0.85 (0.69, 1.05) | 0.022 | 0.93 (0.87, 0.99) |
| Model 1 + Clinical variables‡ | 1.00 (ref.) | 0.98 (0.81, 1.20) | 0.93 (0.75, 1.15) | 0.88 (0.71, 1.10) | 0.94 (0.75, 1.18) | 0.38 | 0.96 (0.90, 1.03) |
| Model 1 + Clinical variables + BMI & WC | 1.00 (ref.) | 1.04 (0.85, 1.28) | 1.02 (0.82, 1.26) | 0.95 (0.76, 1.18) | 1.05 (0.83, 1.31) | 0.98 | 0.99 (0.93, 1.07) |
| Excluding alcohol from MD component |  |  |  |  |  |  |  |
| Model 1 + Alcohol | 1.00 (ref.) | 0.95 (0.78, 1.15) | 0.81 (0.67, 0.99) | 0.85 (0.69, 1.04) | 0.76 (0.61, 0.94) | 0.006 | 0.91 (0.85, 0.97) |
| Model 1 + Alcohol + BMI | 1.00 (ref.) | 1.01 (0.84, 1.23) | 0.91 (0.74, 1.11) | 0.88 (0.71, 1.07) | 0.91 (0.73, 1.13) | 0.17 | 0.95 (0.89, 1.02) |
| Excluding participants with BMI≥30‖ |  |  |  |  |  |  |  |
| Model 1 | 1.00 (ref.) | 1.18 (0.94, 1.49) | 0.95 (0.74, 1.22) | 0.84 (0.65, 1.10) | 0.86 (0.65, 1.13) | 0.037 | 0.92 (0.85, 1.00) |
| Model 1 + BMI | 1.00 (ref.) | 1.17 (0.93, 1.48) | 0.96 (0.75, 1.24) | 0.87 (0.67, 1.13) | 0.98 (0.75, 1.29) | 0.27 | 0.95 (0.88, 1.04) |
| Excluding participants with excessive alcohol consumption** |  |  |  |  |  |  |  |
| Model 1 | 1.00 (ref.) | 1.05 (0.87, 1.28) | 0.86 (0.70, 1.06) | 0.85 (0.69, 1.05) | 0.86 (0.70, 1.08) | 0.041 | 0.93 (0.87, 1.000) |
| Model 1 + BMI | 1.00 (ref.) | 1.09 (0.89, 1.33) | 0.94 (0.76, 1.16) | 0.91 (0.73, 1.13) | 1.04 (0.83, 1.30) | 0.68 | 0.98 (0.92, 1.06) |
| Including participants with probable implausible energy intake†† |  |  |  |  |  |  |  |
| Model 1 | 1.00 (ref.) | 1.03 (0.85, 1.24) | 0.84 (0.69, 1.03) | 0.79 (0.64, 0.97) | 0.85 (0.69, 1.05) | 0.014 | 0.92 (0.86, 0.99) |
| Model 1 + BMI | 1.00 (ref.) | 1.07 (0.89, 1.30) | 0.92 (0.76, 1.13) | 0.86 (0.70, 1.05) | 0.99 (0.80, 1.22) | 0.30 | 0.97 (0.91, 1.04) |
| Excluding participants with secondary causes of hepatic steatosis‡‡ |  |  |  |  |  |  |  |
| Model 1 | 1.00 (ref.) | 1.02 (0.85, 1.24) | 0.84 (0.69, 1.03) | 0.81 (0.65, 1.00) | 0.85 (0.68, 1.05) | 0.021 | 0.92 (0.86, 0.99) |
| Model 1 + BMI | 1.00 (ref.) | 1.08 (0.89, 1.31) | 0.92 (0.75, 1.13) | 0.88 (0.71, 1.09) | 1.02 (0.82, 1.27) | 0.52 | 0.98 (0.91, 1.05) |
| Including participants with diabetes¶¶ |  |  |  |  |  |  |  |
| Model 1 | 1.00 (ref.) | 1.06 (0.9, 1.25) | 0.92 (0.77, 1.09) | 0.84 (0.7, 1.01) | 0.88 (0.73, 1.06) | 0.020 | 0.94 (0.89, 1.00) |
| Model 1 + BMI | 1.00 (ref.) | 1.11 (0.94, 1.31) | 0.94 (0.79, 1.12) | 0.90 (0.75, 1.07) | 1.00 (0.83, 1.21) | 0.32 | 0.97 (0.92, 1.03) |

Abbreviations: FLI, fatty liver index; SES, socio economic status; BMI, body mass index; WC, waist circumference; T2D, type 2 diabetes; MetS, metabolic syndrome; MD,

Mediterranean Diet; NAFLD, non-alcoholic fatty liver disease.

* In categorical analysis, the population was divided into five groups by quintiles (Q1-Q5) of the Mediterranean diet score, Standard deviation was 1.43 and 1.24 for different multivariable analyses, 1.41 and 1.41 after excluding alcohol from Mediterranean Diet score components, 1.42 and 1.23 after excluding participant with excessive alcohol consumption, 1.43 and 1.24 after including participant with probable implausible energy intake or after excluding participant with secondary causes of hepatic steatosis, in the Fenland and CoLaus studies, respectively.

† Adjusted for age (years), sex, marital status (single, married/cohabitant, and divorced/widowed), occupational status (routine and professional jobs in the Fenland study and working and not working in the CoLaus study), education level (compulsory, secondary, and university), smoking status (never, former, and current), energy intake (kcal/day), physical activity energy expenditure (kcal/d, in the Fenland study), total energy expenditure (kcal/day, in the CoLaus study), and date of dietary assessment (in the CoLaus study).

‡ Further adjusted for family history of diabetes (yes/no), high blood pressure (yes/no), high triglyceride level (yes/no), low HDL level (yes/no), and high glucose level (yes/no).

§ Further adjusted for family history of diabetes (yes/no), and metabolic syndrome (yes/no; defined based on International Diabetes Federation)

‖ Excluded 1849 and 541 participants with BMI≥30 kg/m^2^ in Fenland (n=7796) and CoLaus (n=3416), respectively.

** Excessive alcohol consumption defined as >21 units per week for men and >14 units per weeks for women; excluded 2114 and 128 participants with excess alcohol consumption in Fenland (n=7531) and CoLaus (n=3829), respectively.

†† Implausible energy intake defined as <500 and <800 kcal or >3500 or >4000 kcal in women and men, respectively; included 178 and 98 participants with probable implausible energy intake in Fenland (n=9823) and CoLaus (n=4052), respectively.

‡‡ Secondary causes of hepatic steatosis defined as having hepatitis B, C or HIV, and with hepatotoxic medications (Glucocorticoids, isoniazid, methotrexate, amiodarone, and tamoxifen); excluded 117 and 71 participants with probable secondary causes of hepatic steatosis in the Fenland (n=9528) and CoLaus studies (n=3886), respectively.

§§ Calculated based on an algorithm including body mass index, waist circumference, triglycerides, and gamma-glutamyl transferase.

†† Calculated based on an algorithm including presence of the metabolic syndrome and type 2 diabetes, and concentrations of fasting serum insulin, fasting serum aspartate-aminotransferase (AST), and the AST/alanine-aminotransferase ratio.

¶¶ Diabetes defined as glycated haemoglobin ≥48 mmol/mol and/or plasma glucose ≥7.0 mmol/L and/or 2-hour glucose≥11.1 mmol/L with or without presence of glucose lowering drugs or insulin; excluded 418 participants with diabetes from CoLaus (n=4375).

**Table S6** Association between adherence to the Mediterranean diet and prevalence of hepatic steatosis, Fenland Study.

|  | Prevalence ratio (95% CI) across quintiles of pyramid-based Mediterranean diet score* | | | | | *P*-trend | Prevalence ration  (95% CI)  Per SD difference* |
| --- | --- | --- | --- | --- | --- | --- | --- |
|  | Q1 | Q2 | Q3 | Q4 | Q5 |  |  |
| Fenland study, *range* | 3.30-7.84 | 7.85-8.73 | 8.74-9.46 | 9.47-10.28 | 10.29-14.03 |  |  |
| N total | 1929 | 1929 | 1929 | 1929 | 1929 |  |  |
| Normal compare with mild, moderate & severe† |  |  |  |  |  |  |  |
| N cases (score≥5 of 3 to 12) | 602 | 547 | 450 | 391 | 313 |  |  |
| Multivariable + SES + Dietary factor‡ | 1.00 (ref.) | 0.99 (0.86, 1.12) | 0.82 (0.71, 0.95) | 0.76 (0.65, 0.88) | 0.67 (0.56, 0.78) | <0.001 | 0.86 (0.81, 0.90) |
| Multivariable + BMI§ | 1.00 (ref.) | 1.02 (0.89, 1.16) | 0.94 (0.81, 1.08) | 0.90 (0.77, 1.04) | 0.88 (0.75, 1.04) | 0.043 | 0.95 (0.90, 1.00) |
|  |  |  |  |  |  |  |  |
| Normal-mild compare with Moderate-severe‖ |  |  |  |  |  |  |  |
| N cases (score≥7 of 3 to 12) | 59 | 69 | 53 | 32 | 23 |  |  |
| Multivariable + SES + Dietary factor‡ | 1.00 (ref.) | 1.48 (0.97, 2.25) | 1.37 (0.88, 2.13) | 0.69 (0.40, 1.20) | 0.52 (0.27, 0.99) | 0.004 | 0.78 (0.66, 0.93) |
| Multivariable + BMI§ | 1.00 (ref.) | 1.69 (1.10, 2.60) | 2.02 (1.29, 3.17) | 1.06 (0.61, 1.85) | 0.94 (0.49, 1.79) | 0.41 | 0.97 (0.82, 1.15) |
|  |  |  |  |  |  |  |  |
| Ultrasound liver fat score (continuous) |  |  |  |  |  |  |  |
| *mean ± SD* | 4.44 ± 1.20 | 4.38 ± 1.22 | 4.21 ± 1.13 | 4.12 ± 1.03 | 4.00 ± 0.95 |  |  |
| Multivariable + SES + Dietary factor‡ | 1.00 (ref.) | 1.02 (0.96, 1.08) | 0.92 (0.86, 0.98) | 0.88 (0.82, 0.94) | 0.82 (0.76, 0.88) | <0.001 | 0.92 (0.90, 0.94) |
| Multivariable + BMI§ | 1.00 (ref.) | 1.03 (0.97, 1.10) | 0.98 (0.92, 1.05) | 0.97 (0.90, 1.03) | 0.96 (0.90, 1.03) | 0.091 | 0.98 (0.96, 1.003) |

Abbreviations: SES, socio economic status; BMI, body mass index.

* In categorical analysis, the population was divided into five groups by quintiles (Q1-Q5) of the Mediterranean diet score, Standard deviation is 1.43 for pyramid-based Mediterranean diet score.

† Assessed by abdominal ultrasound. The primarily model.

‡ Adjusted for age (years), sex, marital status (single, married/cohabitant, and divorced/widowed), occupational status (routine and professional jobs), education level (compulsory, secondary, and university), smoking status (never, former, and current), energy intake (kcal/day), and physical activity energy expenditure (kcal/d).

§ Further adjusted for BMI.

‖ Ultrasound liver fat score was evaluated as a binary variable with different cut-off points (normal or mild, score ≤7 and moderate or severe, score>7).

** Assessed by abdominal ultrasound. Ultrasound liver fat score was evaluated as a continuous

**Table S7** Association between adherence to the Mediterranean diet and ALT and GGT, Fenland and CoLaus studies.

|  | β coefficient (95% CI) across quintiles of pyramid-based Mediterranean diet score* | | | | | *P*-trend | Prevalence ratio  (95% CI)  Per SD difference* |
| --- | --- | --- | --- | --- | --- | --- | --- |
|  | Q1 | Q2 | Q3 | Q4 | Q5 |  |  |
| Fenland Study, *range* | 3.30-7.84 | 7.85-8.73 | 8.74-9.46 | 9.47-10.28 | 10.29-14.03 |  |  |
| N total | 1,929 | 1,929 | 1,929 | 1,929 | 1,929 |  |  |
| ALT, *median (interquartile range)* | 27 (20, 37) | 25 (19, 35) | 24 (18, 33) | 23 (18, 32) | 22 (17, 30) |  |  |
| Multivariable + SES + Dietary factor† | 1.00 (ref.) | 1.00 (0.96, 1.04) | 0.99 (0.95, 1.04) | 0.99 (0.95, 1.03) | 0.99 (0.95, 1.03) | 0.55 | 0.99 (0.98, 1.01) |
| Multivariable + BMI‡ | 1.00 (ref.) | 1.00 (0.96, 1.04) | 1.00 (0.96, 1.04) | 1.00 (0.96, 1.04) | 1.00 (0.96, 1.04) | 0.96 | 1.00 (0.99, 1.01) |
|  |  |  |  |  |  |  |  |
| GGT, *median (interquartile range)* | 30 (22, 43) | 27 (20, 40) | 25 (20, 36) | 25 (19, 35) | 23 (18, 31) |  |  |
| Multivariable + SES + Dietary factor† | 1.00 (ref.) | 0.99 (0.96, 1.03) | 0.98 (0.94, 1.02) | 0.98 (0.94, 1.02) | 0.96 (0.92, 1.00) | 0.058 | 0.99 (0.97, 0.99) |
| Multivariable + BMI‡ | 1.00 (ref.) | 0.99 (0.96, 1.03) | 0.99 (0.95, 1.03) | 0.99 (0.95, 1.03) | 0.97 (0.93, 1.01) | 0.20 | 0.99 (0.98, 1.004) |
|  |  |  |  |  |  |  |  |
| CoLaus Study, *range* | 1.83-7.45 | 7.46-8.18 | 8.19-8.82 | 8.83-9.47 | 9.48-12.18 |  |  |
| N total | 792 | 791 | 792 | 791 | 791 |  |  |
| ALT, *median (interquartile range)* | 23.5 (18, 34) | 23 (17, 31) | 22 (17, 30) | 22 (17, 29) | 22 (17, 28) |  |  |
| Multivariable + SES + Dietary factor† | 1.00 (ref.) | 1.00 (0.94, 1.06) | 1.00 (0.94, 1.06) | 0.99 (0.93, 1.05) | 0.99 (0.93, 1.05) | 0.66 | 1.00 (0.98, 1.02) |
| Multivariable + BMI‡ | 1.00 (ref.) | 1.00 (0.94, 1.06) | 1.00 (0.95, 1.06) | 0.99 (0.93, 1.05) | 1.00 (0.94, 1.06) | 0.82 | 1.00 (0.98, 1.02) |
|  |  |  |  |  |  |  |  |
| GGT, *median (interquartile range)* | 27 (19, 48) | 25 (18, 39) | 23 (16, 34) | 22 (16, 33) | 20 (15, 31) |  |  |
| Multivariable + SES + Dietary factor† | 1.00 (ref.) | 0.98 (0.93, 1.04) | 0.97 (0.91, 1.03) | 0.96 (0.91, 1.02) | 0.95 (0.90, 1.01) | 0.086 | 0.98 (0.97, 1.002) |
| Multivariable + BMI‡ | 1.00 (ref.) | 0.99 (0.93, 1.04) | 0.97 (0.92, 1.03) | 0.97 (0.91, 1.02) | 0.96 (0.91, 1.02) | 0.14 | 0.99 (0.97, 1.004) |

Abbreviations: ALT, Alanine transaminase; GGT, gamma-glutamyl transferase; SES, socio economic status; BMI, body mass index.

* In categorical analysis, the population was divided into five groups by quintiles (Q1-Q5) of the Mediterranean diet score, Standard deviation is 1.43 and 1.24 for pyramid-based Mediterranean diet score, in the Fenland and CoLaus study, respectively.

† Adjusted for age (years), sex, marital status (single, married/cohabitant, and divorced/widowed), occupational status (routine and professional jobs in the Fenland study and working and not working in the CoLaus study), education level (compulsory, secondary, and university), smoking status (never, former, and current), energy intake (kcal/day), physical activity energy expenditure (kcal/d, in the Fenland study), total energy expenditure (kcal/day-in the CoLaus study), and date of dietary assessment (in the CoLaus study).

‡ Further adjusted for BMI.

# **Table S8** Association between adherence to the Mediterranean diet and prevalence of hepatic steatosis within BMI strata, Fenland and CoLaus studies.

|  |  | Prevalence ratio (95% CI) across quintiles of pyramid-based Mediterranean diet score* | | | | | *P*-trend | Prevalence ratio (95% CI)  per SD difference* |
| --- | --- | --- | --- | --- | --- | --- | --- | --- |
|  |  | Q1 | Q2 | Q3 | Q4 | Q5 |  |  |
| Fenland Study, *range* | | 3.30-7.84 | 7.85-8.73 | 8.74-9.46 | 9.47-10.28 | 10.29-14.03 |  |  |
| N Total | | 1929 | 1929 | 1929 | 1929 | 1929 |  |  |
|  | Ultrasound liver fat score† |  |  |  |  |  |  |  |
| Underweight/Normal | N Cases/total (score≥5 of 3 to 12) | 56/594 | 43/677 | 48/790 | 54/855 | 49/1048 |  |  |
|  | Multivariable + SES + Dietary factor‡ | 1.00 (ref.) | 0.83 (0.53, 1.30) | 0.79 (0.51, 1.25) | 0.83 (0.53, 1.31) | 0.68 (0.42, 1.09) | 0.16 | 0.89 (0.76, 1.03) |
| Overweight | N Cases/total (score≥5 of 3 to 12) | 263/860 | 233/812 | 181/760 | 162/761 | 137/639 |  |  |
|  | Multivariable + SES + Dietary factor‡ | 1.00 (ref.) | 0.98 (0.80, 1.19) | 0.84 (0.67, 1.04) | 0.79 (0.63, 1.00) | 0.91 (0.71, 1.15) | 0.14 | 0.93 (0.86, 1.01) |
| Obese | N Cases/total (score≥5 of 3 to 12) | 283/475 | 271/440 | 221/379 | 175/313 | 127/242 |  |  |
|  | Multivariable + SES + Dietary factor‡ | 1.00 (ref.) | 1.09 (0.90, 1.32) | 0.96 (0.78, 1.18) | 0.93 (0.74, 1.16) | 0.87 (0.67, 1.12) | 0.12 | 0.94 (0.87, 1.02) |
|  |  |  |  |  |  |  |  |  |
|  | FLI§ |  |  |  |  |  |  |  |
| Underweight/Normal | N Cases/total (score≥60) | 13/594 | 12/677 | 10/790 | 11/855 | 2/1048 |  |  |
|  | Multivariable + SES + Dietary factor‡ | 1.00 (ref.) | 1.78 (0.68, 4.70) | 1.57 (0.54, 4.62) | 1.82 (0.68, 4.86) | 0.47 (0.09, 2.38) | 0.38 | 0.89 (0.63, 1.25) |
| Overweight | N Cases/total (score≥60) | 325/860 | 256/812 | 181/760 | 161/761 | 104/639 |  |  |
|  | Multivariable + SES + Dietary factor‡ | 1.00 (ref.) | 0.96 (0.79, 1.15) | 0.83 (0.68, 1.02) | 0.75 (0.60, 0.93) | 0.61 (0.47, 0.78) | <0.001 | 0.85 (0.78, 0.91) |
| Obese | N Cases/total (score≥60) | 435/475 | 364/440 | 305/379 | 252/313 | 179/242 |  |  |
|  | Multivariable + SES + Dietary factor‡ | 1.00 (ref.) | 0.98 (0.83, 1.14) | 0.94 (0.79, 1.12) | 0.98 (0.82, 1.18) | 0.89 (0.72, 1.11) | 0.36 | 0.97 (0.91, 1.04) |
|  |  |  |  |  |  |  |  |  |
| CoLaus Study, *range* | | 1.83-7.45 | 7.46-8.18 | 8.19-8.82 | 8.83-9.47 | 9.48-12.18 |  |  |
| N Total | | 792 | 791 | 792 | 791 | 791 |  |  |
|  | FLI§ |  |  |  |  |  |  |  |
| Underweight/Normal | N Cases/total (score≥60) | 21/323 | 11/359 | 7/367 | 3/401 | 3/445 |  |  |
|  | Multivariable + SES + Dietary factor‡ | 1.00 (ref.) | 0.80 (0.35, 1.82) | 0.68 (0.27, 1.73) | 0.26 (0.07, 0.95) | 0.17 (0.04, 0.78) | 0.006 | 0.64 (0.46, 0.88) |
| Overweight | N Cases/total (score≥60) | 137/325 | 123/315 | 82/329 | 67/281 | 72/271 |  |  |
|  | Multivariable + SES + Dietary factor‡ | 1.00 (ref.) | 1.04 (0.80, 1.36) | 0.74 (0.55, 0.99) | 0.69 (0.51, 0.95) | 0.81 (0.59, 1.11) | 0.023 | 0.89 (0.81, 0.98) |
| Obese | N Cases/total (score≥60) | 139/144 | 106/117 | 81/96 | 90/109 | 60/75 |  |  |
|  | Multivariable + SES + Dietary factor‡ | 1.00 (ref.) | 0.99 (0.74, 1.31) | 0.90 (0.67, 1.22) | 0.91 (0.68, 1.22) | 0.88 (0.63, 1.22) | 0.35 | 0.95 (0.86, 1.05) |
|  |  |  |  |  |  |  |  |  |
|  | NAFLD liver fat score‖ |  |  |  |  |  |  |  |
| Underweight/Normal | N Cases/total (score≥-0.640) | 29/323 | 34/359 | 24/367 | 16/401 | 23/445 |  |  |
|  | Multivariable + SES + Dietary factor‡ | 1.00 (ref.) | 1.34 (0.79, 2.28) | 0.91 (0.51, 1.64) | 0.55 (0.28, 1.07) | 0.80 (0.43, 1.46) | 0.056 | 0.86 (0.72, 1.04) |
| Overweight | N Cases/total (score≥-0.640) | 124/325 | 132/315 | 131/554 | 88/281 | 92/271 |  |  |
|  | Multivariable + SES + Dietary factor‡ | 1.00 (ref.) | 1.12 (0.86, 1.46) | 0.90 (0.68, 1.19) | 0.94 (0.70, 1.25) | 1.02 (0.76, 1.38) | 0.69 | 0.98 (0.89, 1.08) |
| Obese | N Cases/total (score≥-0.640) | 104/144 | 74/117 | 59/96 | 64/109 | 57/75 |  |  |
|  | Multivariable + SES + Dietary factor‡ | 1.00 (ref.) | 0.98 (0.7, 1.37) | 0.86 (0.61, 1.22) | 0.88 (0.63, 1.24) | 1.20 (0.84, 1.71) | 0.52 | 1.01 (0.90, 1.14) |

Abbreviations: FLI, fatty liver index; SES, socio economic status; BMI, body mass index; NAFLD, non-alcoholic fatty liver disease; NE, not estimated.

Body mass index categorized as “underweight/normal” (BMI of <25), “overweight” (BMI of 25-<30), and “obese” (BMI of ≥30).

* In categorical analysis, the population was divided into five groups by quintiles (Q1-Q5) of the Mediterranean diet score, Standard deviation is 1.43 and 1.24 for pyramid-based Mediterranean diet score, in the Fenland and CoLaus studies, respectively.

† Assessed by abdominal ultrasound.

‡ Adjusted for age (years), sex, marital status (single, married/cohabitant, and divorced/widowed), occupational status (routine and professional jobs in the Fenland study and working and not working in the CoLaus study), education level (compulsory, secondary, and university), smoking status (never, former, and current), energy intake (kcal/day), physical activity energy expenditure (kcal/d, in the Fenland study), total energy expenditure (kcal/day, in the CoLaus study), and date of dietary assessment (in the CoLaus study).

§ Calculated based on an algorithm including body mass index, waist circumference, triglycerides, and gamma-glutamyl transferase.

‖ Calculated based on an algorithm including presence of the metabolic syndrome and type 2 diabetes, and concentrations of fasting serum insulin, fasting serum aspartate-aminotransferase (AST), and the AST/alanine-aminotransferase ratio.
